# Quantitative differentiation of benign and misfolded glaucoma-causing myocilin variants on the basis of protein thermal stability

**DOI:** 10.1242/dmm.049816

**Published:** 2023-01-13

**Authors:** Hailee F. Scelsi, Kamisha R. Hill, Brett M. Barlow, Mackenzie D. Martin, Raquel L. Lieberman

**Affiliations:** School of Chemistry & Biochemistry, Georgia Institute of Technology, 901 Atlantic Dr. NW, Atlanta, GA 30332-0400, USA

**Keywords:** Glaucoma, Misfolding, Genetic mutation, Protein homeostasis, Thermal stability

## Abstract

Accurate predictions of the pathogenicity of mutations associated with genetic diseases are key to the success of precision medicine. Inherited missense mutations in the myocilin (*MYOC*) gene, within its olfactomedin (OLF) domain, constitute the strongest genetic link to primary open-angle glaucoma via a toxic gain of function, and thus *MYOC* is an attractive precision-medicine target. However, not all mutations in *MYOC* cause glaucoma, and common variants are expected to be neutral polymorphisms. The Genome Aggregation Database (gnomAD) lists ∼100 missense variants documented within OLF, all of which are relatively rare (allele frequency <0.001%) and nearly all are of unknown pathogenicity. To distinguish disease-causing OLF variants from benign OLF variants, we first characterized the most prevalent population-based variants using a suite of cellular and biophysical assays, and identified two variants with features of aggregation-prone familial disease variants. Next, we considered all available biochemical and clinical data to demonstrate that pathogenic and benign variants can be differentiated statistically based on a single metric: the thermal stability of OLF. Our results motivate genotyping *MYOC* in patients for clinical monitoring of this widespread, painless and irreversible ocular disease.

## Teaser

**Summary:** Differentiating benign from misfolding, glaucoma-associated mutations in the myocilin olfactomedin domain has been a challenge. We show that the pathogenicity of myocilin variants can be predicted by a thermal stability cutoff of 47°C.

## INTRODUCTION

Knowledge of how genotype variations lead to phenotypic effects increases our understanding of genomic contributions to disease and can allow us to tailor clinical care (Ashley, 2016). However, the physiological consequences of single-nucleotide substitutions leading to changes in coding sequence are often not straightforward to predict. Predicting phenotype from genotype is particularly challenging in situations in which there is a toxic gain of function (Flanagan et al., 2010), such as protein aggregation seen in myocilin-associated glaucoma. Non-synonymous monoallelic coding mutations in the myocilin gene (*MYOC*, GenBank accession NM_000261.2), predominantly within its olfactomedin (OLF) domain (Resch and Fautsch, 2009), cause early-onset open-angle glaucoma (OAG). Myocilin has no known function (Resch and Fautsch, 2009), and there is no glaucoma phenotype in *Myoc* knockout mice (Gould et al., 2006; Kim et al., 2001) or in individuals with a homozygous N-terminal truncation mutation (Lam et al., 2000) or hemizygous deletion of *MYOC* (Wiggs and Vollrath, 2001), but rare non-synonymous coding mutations lead to toxic intracellular sequestration of misfolded myocilin (Jacobson et al., 2001; Joe et al., 2003; Yam et al., 2007). Currently, in the absence of clinical details, a multigenerational familial inheritance pattern and supporting laboratory data, the pathogenicity of any given mutation is not definitive (Scelsi et al., 2021) because recent genome-wide association studies demonstrate that mutations in *MYOC* in general are not associated with glaucoma (Craig et al., 2020). Therefore, even though OAG afflicts approximately 70 million individuals worldwide (Allison et al., 2020), myocilin variants that are found frequently in the general population are expected to be benign polymorphisms.

Estimates suggest that myocilin-associated glaucoma accounts for 3-4% of OAG cases (Resch and Fautsch, 2009). Initially, familial genetics studies identified a rare OLF mutation with a dominant inheritance pattern alongside detailed clinical data (Stone et al., 1997) and, soon thereafter, a case–control study of glaucoma patients and age-matched controls identified probable glaucoma-causing myocilin variants (Fingert et al., 1999). However, over the course of the last 20 years, some myocilin mutations that once were only found in glaucoma patients emerged from large-scale genome-sequencing projects, such as those collated in the Genome Aggregation Database (gnomAD, https://gnomad.broadinstitute.org/) (Karczewski et al., 2020), raising questions about the likelihood of their pathogenicity. More than 200 non-synonymous variants resulting from single nucleotide substitutions, ∼100 within OLF (Table 1; Table S1), currently appear in gnomAD. Still, even the most prevalent OLF-resident myocilin variants are relatively rare, as their overall frequency is nearly 100-fold lower than the most frequent genetic variation in myocilin, R76K (0.1% allele frequency). Illustrative of the inherent challenges in identifying disease variants in the case of a toxic gain of function like myocilin-associated glaucoma, there are often conflicting annotations of pathogenicity in the literature and in gnomAD. More generally, it is challenging to predict pathogenicity based on physicochemical compatibility of a given OLF substitution (Hill et al., 2019b) and, for the majority of population variants (Table 1; Table S1), clinical data are completely absent.

**Table 1.**
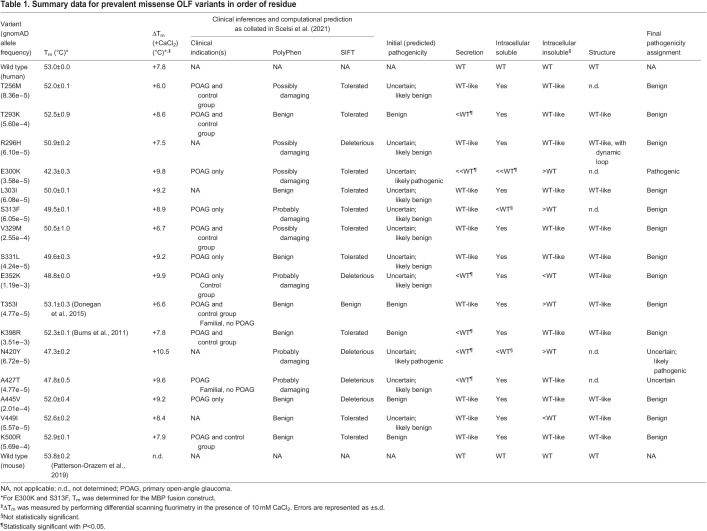
Summary data for prevalent missense OLF variants in order of residue

**Fig. 1.**
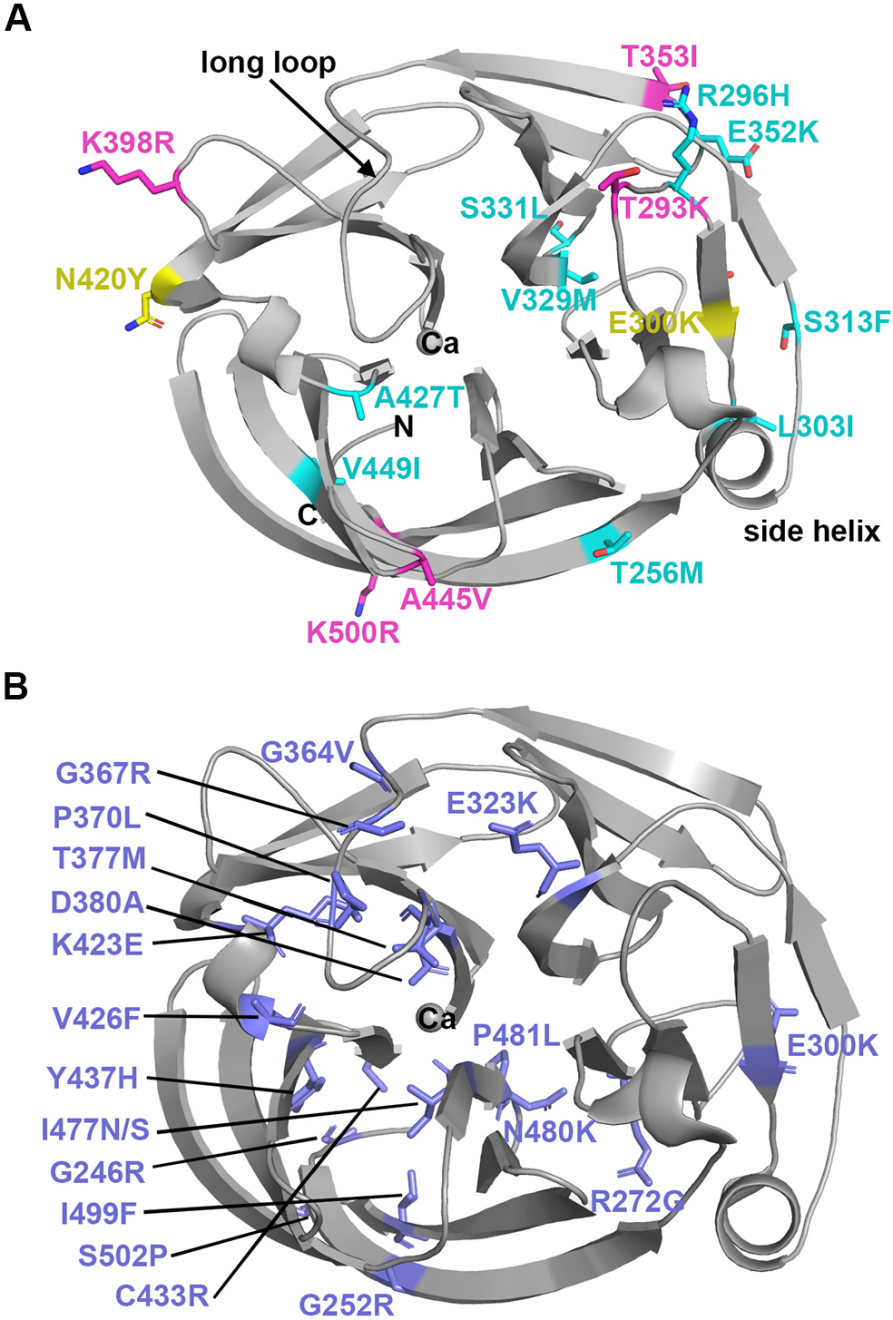
**Myocilin variants considered in this study mapped on the crystal structure of the OLF domain.** (A) Pathogenicity predictions for 16 myocilin variants. Predicted benign variants are indicated in magenta, uncertain variants that are likely benign are in cyan and uncertain variants that are likely pathogenic are in yellow. (B) Pathogenic variants included in analyses conducted in this study (Fig. 5 analysis; Fig. S5). Two other pathogenic variants, P370L and W286R, were excluded from analysis because no monomeric OLF could be purified for these variants and thus a T_m_ could not be measured.

Here, we first tested the hypothesis that common variants are polymorphisms by biochemically evaluating the 16 most frequently reported OLF-resident myocilin risk alleles (Fig. 1A; Table 1; allele count of ten or higher) for multiple observable features associated with myocilin misfolding and compared these to characteristics of previously characterized misfolding disease variants (Fig. 1B). Given that disease-associated myocilin variants are retained intracellularly as insoluble aggregates instead of being secreted, we evaluated secretion using a cellular assay (Liu and Vollrath, 2004; Zhou and Vollrath, 1999) and visualized intracellular myocilin sequestration using confocal imaging. As purified isolated OLF domain variants exhibit decreased thermal stability (Lieberman and Ma, 2021), we expressed and purified the OLF domains of these variants and characterized them using biophysical methods including thermal stability measurements, circular dichroism (CD) and, where possible, X-ray crystallography. These experiments support the finding that genetic polymorphisms in *MYOC* do not confer glaucoma susceptibility (Craig et al., 2020), but nevertheless demonstrate that misfolding variants are present among documented population variants.

Second, we combined all available clinical and laboratory data on OLF variants to reevaluate how benign variants can be distinguished from those that are pathogenic (Lieberman and Ma, 2021). Among the common features associated with pathogenic myocilin misfolding, we demonstrate that a single metric, OLF thermal stability, can robustly segregate benign from early-onset disease variants. Taken together, our results motivate *MYOC* genotyping and subsequent early clinical monitoring of individuals with susceptible alleles for hallmarks of glaucoma pathogenesis.

## RESULTS

### Predictions of pathogenicity

OLF-resident myocilin variants with allele counts of ten or higher in gnomAD, 16 in total, were chosen for this analysis. To predict pathogenicity for the selected variants (Table 1), we studied multiple sources of data (reviewed in Scelsi et al., 2021), including clinical descriptions available in the literature, computational predictions [using PolyPhen (Adzhubei et al., 2010) and the sorting intolerant from tolerant (SIFT) algorithm (Vaser et al., 2016)], available cellular secretion (Gobeil et al., 2006; Vollrath and Liu, 2006; Zadoo et al., 2016) and detergent insolubility data (Zhou and Vollrath, 1999), existing biophysical data on the isolated OLF domain (Donegan et al., 2012, 2015) and physicochemical analysis based on our OLF structure. At the outset of our study, five variants could be confidently predicted as benign polymorphisms: T293K, T353I, K398R, A445V and K500R. These mutations are all found on the protein surface (Fig. 1A) and well documented in the literature (Scelsi et al., 2021). Three of these variants harbor conservative substitutions (Lys to Arg, Ala to Val). Thr to Lys and Thr to Ile are less conservative changes, but in prior studies, T293K and T353I exhibited wild type (WT)-like cellular secretion and WT-like thermal stability, and were found in the control group for case–control clinical studies (Table 1).

The 12 remaining variants in our study were of uncertain pathogenicity. Overall, nine variants (T256M, R296H, L303I, S313F, V329M, S331L, E352K, A427T, and V499I) were considered likely to be benign (Fig. 1A). V449I, a conservative substitution found in a hydrophobic region of the OLF β-propeller, appears only in gnomAD. The remaining variants have been described in the clinical literature, but there is limited, if any, laboratory-based characterization of these variants, or the available data are not consistent with a confident benign or pathogenic annotation. Illustrative of this latter group is E352K, a variant documented in gnomAD as appearing predominantly in the African or African American population. Charge-inversion variants cause disease in a number of other disorders (Martinsson et al., 2000; Ojeda et al., 2015; Szpiech et al., 2017). E352K has been computationally predicted to be pathogenic and has been documented both in primary open-angle glaucoma (POAG) patients and in control groups (Alward et al., 1998; Fingert et al., 1999; Liu et al., 2012; Whigham et al., 2011), yet exhibits WT-like secretion (Gobeil et al., 2006). Similarly, V329M, a substitution that occurs in the hydrophobic interior of OLF, is not sterically compatible. Previous support for the V329M variant as benign comes primarily from the observation that methionine is the corresponding residue in *Danio rerio* myocilin, but the mutation has also been documented among glaucoma patients (Fingert et al., 1999; Liu et al., 2012; Shimizu et al., 2000; Svidnicki et al., 2018), including one diagnosed with juvenile OAG (Svidnicki et al., 2018). Finally, we considered the remaining two variants, E300K and N420Y, as likely to be pathogenic. The internal charge-inversion variant E300K has only been described twice in the clinical literature (Fan et al., 2005; Pang et al., 2002). To our knowledge, no clinical reports of N420Y have been published, and we reasoned that the polar-to-aromatic change Asn to Tyr on a surface-exposed residue would not be tolerated.

### Cellular secretion assay identifies three myocilin variants – E300K, S313F and N420Y – with defects suggestive of misfolding

**Fig. 2.**
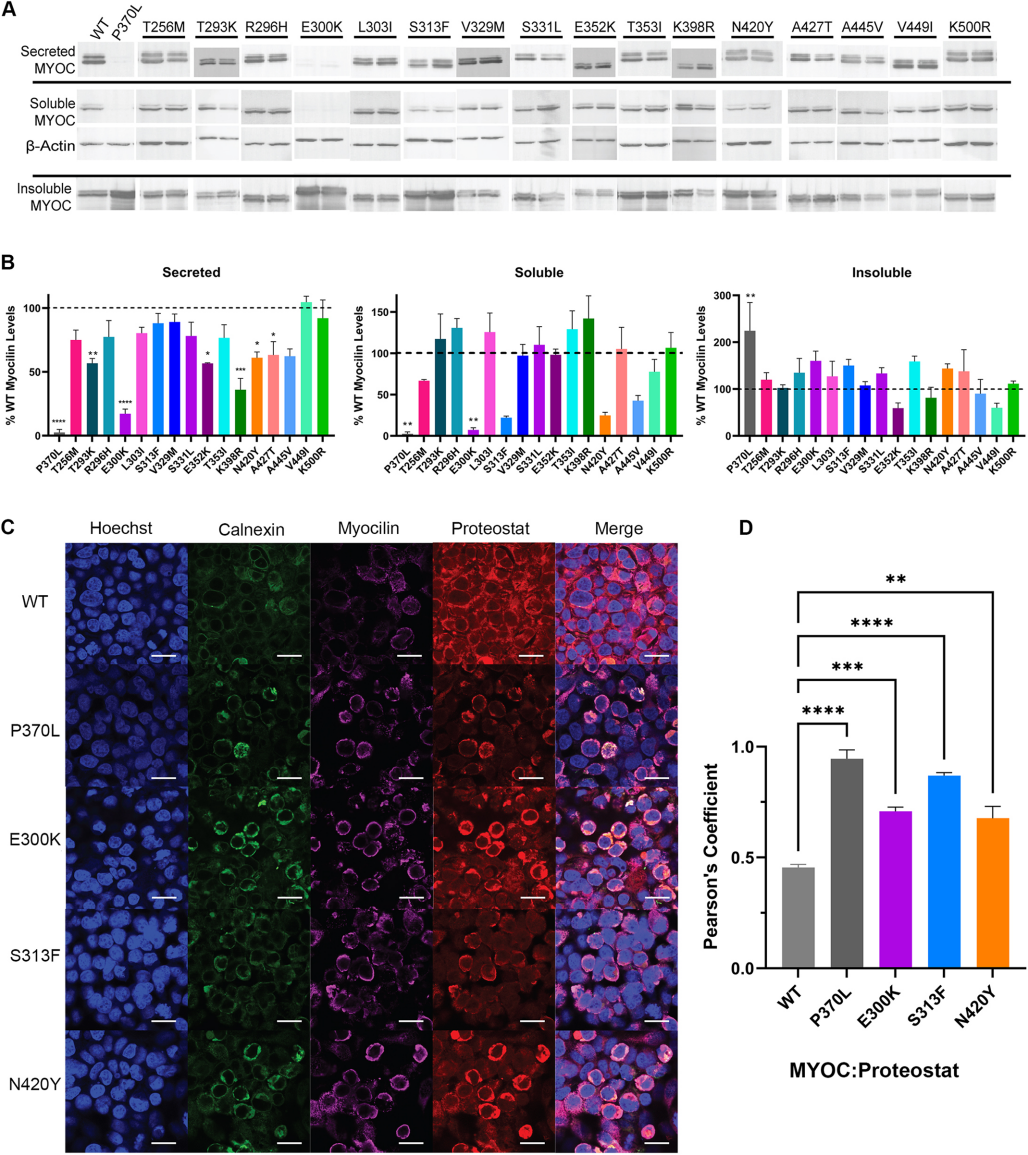
**Analysis of cellular secretion of myocilin variants.** (A) Western blots of secreted, intracellular soluble and intracellular insoluble fractions of HEK293T cells transiently transfected with plasmids encoding MYOC variants that were of interest in this study, presented in analytical duplicate, in residue order. β-actin was used as a loading control for soluble myocilin. Full blots are presented in Fig. S1. Data are representative of two biological replicates. (B) Quantification of the western blots shown in A and their biological replicates (not shown), normalized to WT myocilin (dashed line). (C) Confocal imaging of HEK293T cells transiently transfected with plasmids encoding MYOC variants showing nuclear staining with Hoechst 33342 (blue), ER staining with calnexin (green), myocilin staining (magenta) and amyloid staining (Proteostat, red). Scale bars: 20 µm. See Fig. S2 for images of no-transfection control. (D) Colocalization of myocilin with Proteostat using *n*=3 fields of view within a single technical replicate. For B, D, error bars shown are ±s.e.m., and one-way ANOVA was used to assess statistical significance of variants compared to WT using Tukey's multiple comparisons test for post hoc analysis. *P< 0.05; **P<0.01; ***P<0.001; ****P<0.0001. See additional statistical analysis with no-transfection control in Fig. S2.

To assess the toxic gain of function of myocilin misfolding, we compared the extent of myocilin secretion (Gobeil et al., 2006; Liu and Vollrath, 2004; Vollrath and Liu, 2006) to its intracellular accumulation (Zhou and Vollrath, 1999). We used western blot analysis to qualitatively probe for myocilin in three locations: in spent media and in two intracellular fractions, namely, detergent soluble and detergent insoluble (Vollrath and Liu, 2006; Zhou and Vollrath, 1999). Variants were tested in the assay in no particular order to avoid bias but the western blot results are rearranged in residue order in Fig. 2A. Assessment of spent media from cells expressing each of the 16 full-length myocilin variants revealed WT-like secretion for T256M, R296H, L303I, S313F, V329M, S331L, T353I, A445V, V449I and K500R. Modestly lower levels than WT were detected for T293K, E352K, K398R, N420Y and A427T (Fig. 2A,B; Fig. S1). E300K was secreted to the least extent (Fig. 2A,B; Fig. S1), similar to P370L, consistent with a previous report using a luciferase-based assay (Zadoo et al., 2016). Analysis of the intracellular detergent-soluble fractions revealed E300K to be a statistical outlier among the variants, but T256M, S313F, N420Y, and A445V also presented decreased soluble protein levels compared to those of WT myocilin (Fig. 2A,B). The detergent-insoluble myocilin fractions were highest for E300K, S313F, T353I and N420Y. These variants did not accumulate to a degree statistically higher than WT levels nor to a level significantly lower than the levels of the severe disease variant P370L (Fig. 2A,B; Fig. S1).

Overall, only E300K, S313F and N420Y consistently exhibited defects suggestive of disease-like behavior: intracellular insolubility levels >50% of WT levels, decreased cellular secretion <50% of WT levels and/or intracellular solubility levels >50% of WT levels (Fig. 2A,B). Despite some variability across experiments, no other variants met these criteria, e.g. we excluded T353I due to sufficient secreted and soluble levels, despite it exhibiting elevated levels of insoluble protein. There are numerous challenges with this assay, including lack of loading controls for secreted and insoluble fractions, and difficulty in handling the insoluble fraction for loading into the gel to yield quantitative comparisons.

To complement western blot analysis and further characterize insoluble aggregates produced by cells expressing E300K, S313F and N420Y myocilin, next we stained cells with Proteostat, a dye used to visualize amyloid aggregates (Shen et al., 2011). Previously, we showed that the related molecule thioflavin T stains intracellular aggregates of P370L but not WT myocilin in Chinese hamster ovary cells (Orwig et al., 2012). Intracellular aggregates of E300K, S313F or N420Y appeared as puncta (Fig. 2C) when stained with Proteostat and colocalized with calnexin in the endoplasmic reticulum (ER) (Fig. 2D; Fig. S2), with levels reaching statistical significance over that of WT (Fig. 2D). These results indicate that aggregates of E300K, S313F or N420Y have some amyloid-like character when transiently expressed in HEK293T cells.

### In the context of the isolated OLF domain, four variants – E300K, S313F, N420Y and A427T – exhibit some characteristics suggestive of misfolding

**Fig. 3.**
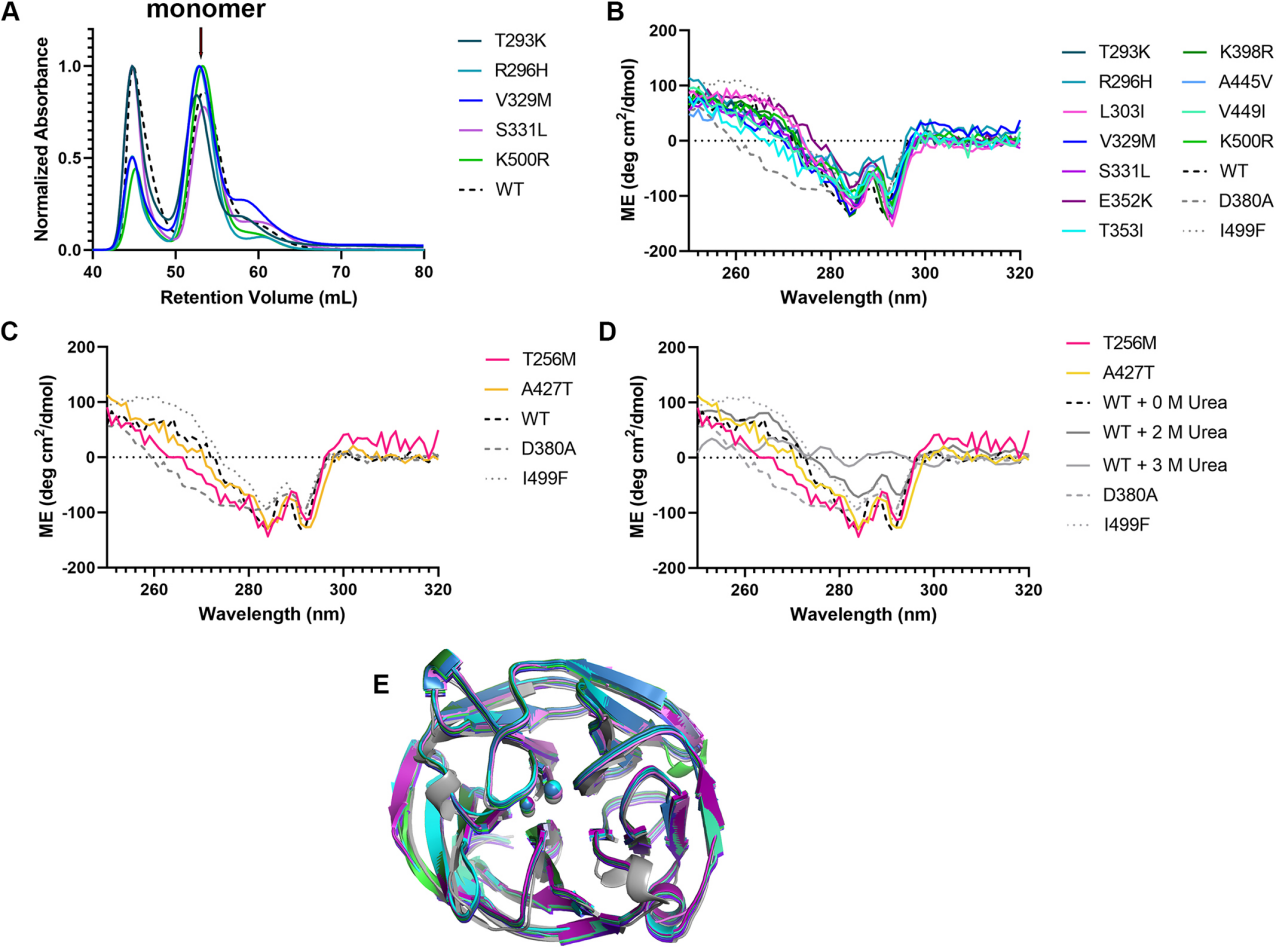
**Biophysical and structural characterization of wild-type-like OLF variants.** (A) SEC profiles highlighting robust production of monomeric MBP-OLF for these variants. (B) Near-UV CD spectra comparing tertiary structure across WT-like variants that were structurally characterized (see E). (C) Near-UV CD spectra comparing tertiary structure across WT-like variants for which structures were not obtained. (D) Comparison of near-UV CD spectra between variants for which structures were not obtained and WT OLF with increasing urea concentration. ME, molar ellipticity. (E) Superposition of 11 crystal structures with that of WT OLF – T293K, R296H, L303I, V329M, S331L, E352K, T353I, K398R, K500R, A445V and V449I. Crystallographic statistics are given in Table S2.

**Fig. 4.**
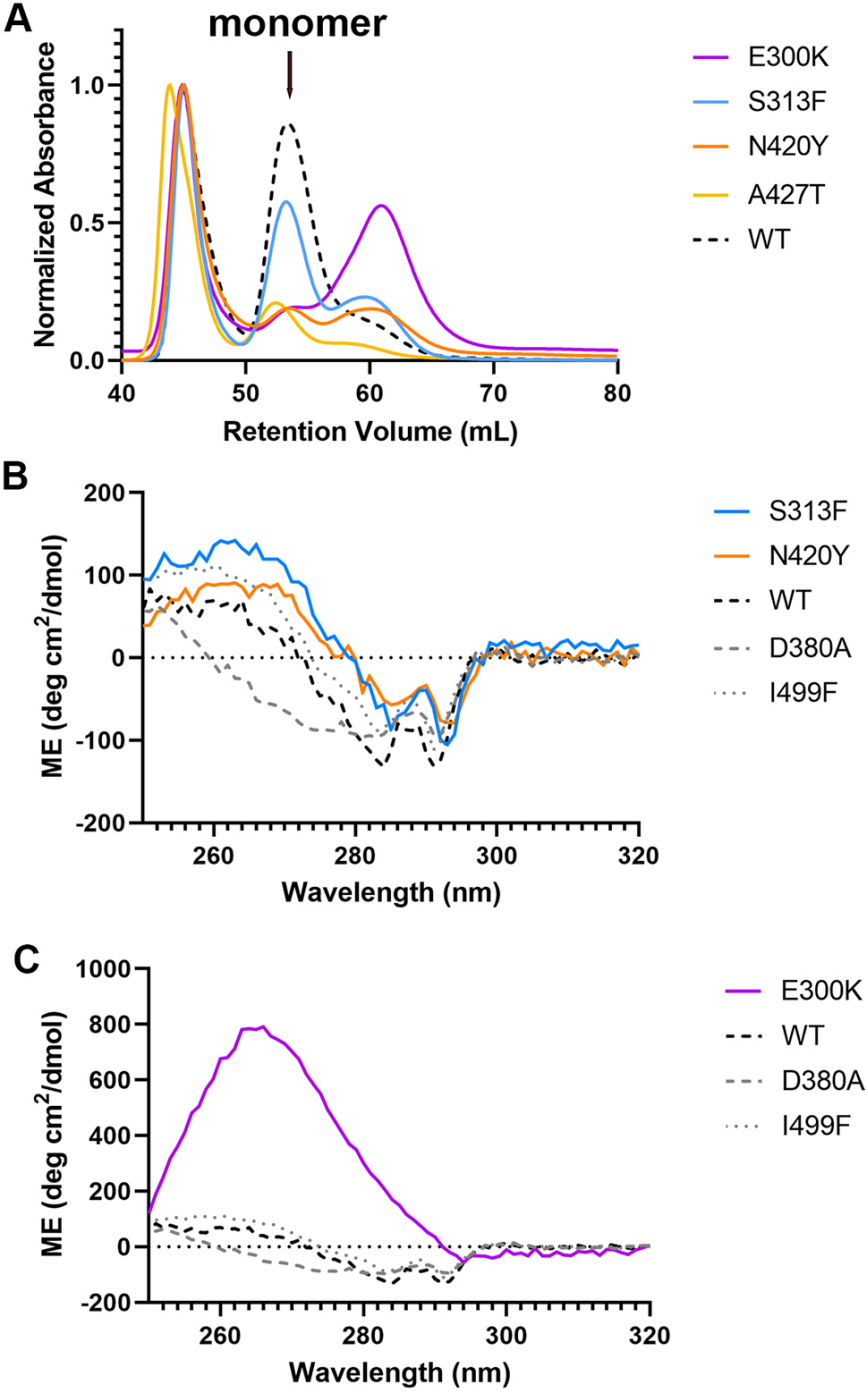
**Biophysical and structural characterization of non-WT-like OLF variants.** (A) SEC profile highlighting limited production of monomeric MBP-OLF for the indicated variants. (B) Near-UV spectra highlighting similar tertiary structure signatures of disease-like variants with that of I499F. (C) Near-UV spectrum of E300K highlighting differences from those of WT, D380A and I499F. ME, molar ellipticity.

For each of the 16 variants tested in the cellular secretion assay, the isolated OLF domain variants were expressed and purified. As an initial gauge of misfolding propensity, we compared the relative *Escherichia coli* expression of the monomeric fusion protein (maltose binding protein-OLF, MBP-OLF) to soluble aggregates, which we fractionated by size-exclusion chromatography (SEC) (Burns et al., 2010). Consistent with the 13 variants for which the secretion profiles were similar to those of WT, the monomer accounted for >40% of the expressed fusion protein of interest (Fig. 3A); A427T was at the lower cutoff (Fig. 4A). After cleavage and isolation of the corresponding OLF variant, each exhibited thermal melting temperatures (T_m_) within ∼4°C of WT, including two variants with T_m_ values higher than for WT (Table 1).

To compare the signatures of tertiary and secondary structural signatures in solution, we next acquired near- and far-ultraviolet (UV) CD spectra, respectively (Fig. 3B-D; Fig. S3). The 13 OLF variants with WT-like T_m_ values had CD spectra that most closely resembled those of WT and differed from the moderately destabilized disease mutant D380A and the severely destabilized disease mutant I499F (Fig. 3B,C). No differences were seen in far-UV spectra, indicating that the secondary structure remained intact (Fig. S3A). For additional reference, we acquired near-UV spectra of WT OLF as a function of urea concentration. A major change in the CD spectrum occurred between 2 and 3[Supplementary-material sup1]M urea (Fig. 3D); at higher urea concentrations, the ellipticity returns to the baseline because the tertiary structure of OLF is lost. Of these 13, only T256M spectra were modestly distinguishable from the spectra of WT in 0-2 M urea (Fig. 3D).

Structural integrity was further confirmed by high-resolution crystal structures, which were solved for 11 OLF variants. No gross changes from WT OLF were detected for the structures of T293K, R296H, L303I, V329M, S331L, E352K, T353I, K398R, A445V, V449I or K500R (Fig. 3E; Fig. S4; Table S2), indicating that these mutations are accommodated within the native five-bladed β-propeller fold and do not perturb the dinuclear calcium-containing site (Donegan et al., 2015) (Fig. 1A). Interestingly, R296H has one loop comprising residues 261-266 that was difficult to model, perhaps because H296 is smaller and adopts two conformations (Fig. S4). Despite extensive effort, T256M and A427T did not crystallize. The tertiary structure of T256M in solution does differ somewhat from WT (Fig. 3C,D), perhaps explaining why it was recalcitrant to crystallization. Why A427T did not crystallize remains unclear but, unlike T256M, A427T also exhibits somewhat decreased stability compared to that of WT (Table 1).

### Twelve variants can be assigned as benign polymorphisms

In addition to the five variants that we predicted initially to be benign (T293K, T353I, K398R, A445V and K500R), our study reveals seven more (T256M, R296H, L303I, V329M, S331L, E352K and V499I) that are indistinguishable from WT OLF across multiple assays. Thus, our data indicate these should also be considered benign. Surprisingly, V329M does not appear to harbor any pathogenic signatures despite its location in an internal strand associated with amyloid formation (Hill et al., 2014) and its involvement in a hydrophobic patch that is disrupted in other disease variants like W286R (Fingert et al., 1999). Similarly, the finding that E352K does not exhibit characteristics of misfolding is consistent with the later age of diagnosis of glaucoma for patients with the E352K variant (Faucher et al., 2002; Williams-Lyn et al., 2000) and a lack of clear Mendelian inheritance pattern (Allingham et al., 1998). Most of the patients with E352K are of African or African American ancestry, a group more prone to developing POAG (Allingham et al., 1998; Rudnicka et al., 2006). Our findings rule out misfolding of E352K as causal for this higher glaucoma risk (Kapetanakis et al., 2016; Rudnicka et al., 2006) and suggest that other mechanisms are likely at play.

### Three variants – E300K, S313F and N420Y – with cellular secretion defects also exhibit characteristics of misfolding *in vitro*

Expression of E300K and N420Y as monomeric MBP-OLF fusion proteins in *E. coli* was notably diminished, suggestive of misfolding, and that of S313F was lower than that of WT, near our designated cutoff (Fig. 4A). The T_m_ values measured for E300K (42.3°C) and N420Y (47.3°C) were lower than that of WT, whereas that of S313F (49.5°C) was close to that of WT OLF, similar to that of *Mus musculus* OLF (Table 1). Far-UV spectra were similar to those of WT, indicating an intact secondary structure (Fig. S3C). The near-UV spectra for N420Y, S313F and E300K diverged from that of WT with a more positive signal in the range of 250 to 295 nm. Whereas the deviations for N420Y and S313F were mild (Fig. 4B), the near-UV CD spectrum of E300K showed marked differences (Fig. 4C), distinct from any species detectable with urea unfolding of WT OLF (Fig. 3D) as well as any glaucoma variants characterized to date. The local environment of E300 includes a cluster of aromatic residues, W286, F299 and Y301, which could contribute to this altered spectrum (Kelly and Price, 2000).

### Disease variants segregate from benign variants by thermal stability

Based on the data presented here, we revisited the correlation between OLF variant stability and disease severity as proxied by clinical ‘age at diagnosis’. This metric is a poor indicator for age of disease onset because glaucoma is a painless disease that goes largely unnoticed until peripheral vision loss is noticed, which occurs at a late stage of the disease. In addition, there is an age at diagnosis for variants now considered benign because individuals harboring myocilin polymorphisms can still develop glaucoma even if the relationship is not causal. Nevertheless, in our early biophysical study of the OLF domain (Burns et al., 2011), a weak positive linear correlation between OLF variant T_m_ and age at diagnosis was suggested, and this apparent relationship was repeated in a recent literature review by others (Tanji et al., 2021). Upon plotting the new variants characterized here alongside all variants for which clinical and experimental stability data are available, no convincing trendline was observed, however (Fig. S5). In sum, with the additional data accrued over time, a linear correlation between age at diagnosis and OLF thermal stability no longer appears credible.

**Fig. 5.**
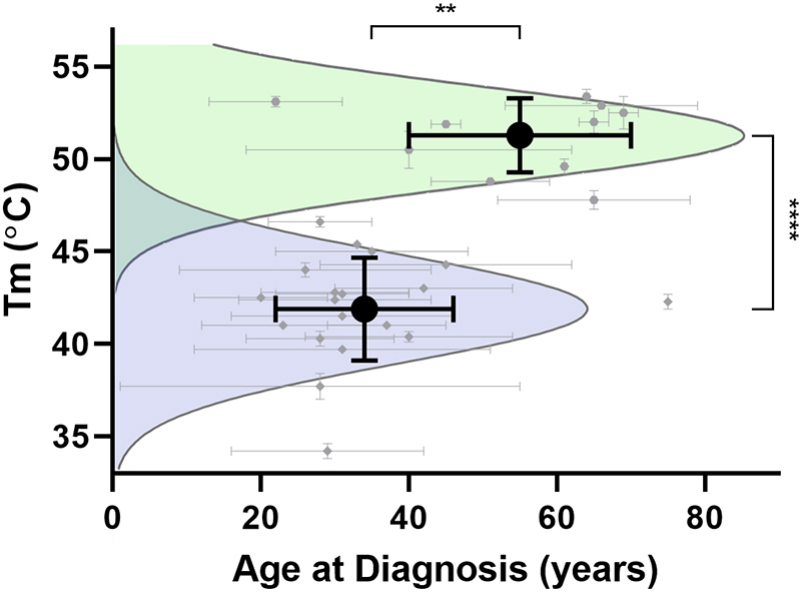
**Correlation between age at diagnosis and thermal stability.** Average age of diagnosis (±s.d.) and average T_m_ (±s.d.) for each cluster (black circle) are statistically significant. Purple and green curves show Gaussian representations of pathogenic and benign variants, respectively. **P=0.01; ****P<0.0001.

We next considered alternative relationships between age at diagnosis and thermal stability (Fig. 5). Using all available data from our laboratory from the past decade, in many cases measured by different individuals and from the clinical literature (Table S3), we show that the data robustly cluster in two ways. First, the mean ages at diagnosis for benign (mean=49, *n*=21) and pathogenic (mean=36, *n*=206) variants are statistically significant (*P*=0.01). Second, and more impressively, the average thermal stability of benign (mean±s.d.=51.4±1.9°C, *n*=50) and disease variants (mean±s.d.=41.7±2.8°C, *n*=63) segregates with *P*<0.0001 (*P*=1.2×10^−40^). All data can be captured between two standard deviations of the mean for benign and pathogenic variants, with a visual cutoff of 47°C.

## DISCUSSION

Thermal stability is an intrinsic property of a protein, dictated by its amino acid sequence, and a single-residue substitution can perturb thermal stability (Dill, 1990), leading to pathogenic consequences. In general, the effect of loss-of-function mutations is more tractable, and thus better understood and predicted, than scenarios in which point variants lead to a toxic gain of function, such as aggregation (Flanagan et al., 2010). In the case of myocilin-associated glaucoma, rare familial variants are highly penetrant. However, because mutations in *MYOC* are not generally causal for glaucoma, pathogenicity is currently considered only with prioritized clinical metrics, and where available, laboratory data, such as that curated by ClinGen (https://www.clinicalgenome.org) and related projects (https://myocilin.com) (Hewitt et al., 2008). Unlike other diseases, glaucoma progresses without pain or visual field symptoms, and currently, regular screening is not indicated for asymptomatic individuals under the age of 40 (Fleming et al., 2005).

This study expands our appreciation for the myriad ways by which coding mutations can unpredictably affect protein structure and lead to pathogenic misfolding, particularly in the case of myocilin OLF. Previously, we established that disease variants within OLF were thermally destabilized (Burns et al., 2011) and exhibited non-native structural signatures in solution (Hill et al., 2014); the other structural domain of myocilin, a coiled coil, does not aggregate and has a T_m_ ∼15°C higher than OLF (67°C; Hill et al., 2017). However, OLF can tolerate particular variations (Hill et al., 2019a; Hill et al., 2019b), including some that lead to pronounced structural deviations (Hill et al., 2019b), without pathogenic signatures. For example, the D478S point mutation is thermally tolerated even though it ablates calcium binding, shifts an entire β-propeller blade by 0.5 Å and disorders the side helix (Fig. 1A) (Hill et al., 2019b). We also generated a computationally-predicted OLF variant with 21 mutations that retains the native structure and is thermostable (Goldenzweig et al., 2016; Hill et al., 2019b), raising the question of why evolution has led to only a moderately stable, misfolding-prone domain for expression in an eye tissue critical to vision.

As genome sequencing information becomes progressively widespread, it is increasingly valuable and possible to accurately predict the pathogenicity of missense variants that cause genetic disease. A major outcome of this study is an apparent thermal stability threshold for disease variants of ∼47°C. That is, variants with OLF stabilities <47°C can confidently be predicted to confer associated secretion defects and intracellular accumulation of insoluble aggregates, consistent with those of bona fide disease variants. The thermal stability difference between benign and pathogenic variants is statistically rigorous, more so than clinical age at diagnosis, owing to its weak correlation with disease onset and severity. Although the secretion assay played a pivotal role in the early days of myocilin research and has been valuable over the years for the characterization of the cellular behavior of mutant myocilin proteins, the assay can be difficult to interpret due to limitations in quantitation. Indeed, in our cellular assay, some misfolding characteristics were initially detected for T353I and S313F but, ultimately, other characteristics including thermal stabilities were found to be within the bounds of benign variants.

Our data indicate that pathogenic variants are present in gnomAD. From our study, E300K and N420Y should be considered likely to be pathogenic because their stabilities fall within the range of those of disease variants and they exhibited other signatures of misfolding. As expected for entries in gnomAD, N420Y has not been reported in the clinical literature, so connections to ocular health are uncertain. E300K was documented once in the literature in a patient of unknown age (Fan et al., 2005) and once in an OAG patient diagnosed at the age of 75 (Pang et al., 2002). Among the remaining entries in gnomAD (Table S1), T377M, the pathogenic variant with a T_m_ of 44.3°C, appears five times. Our study suggests that individuals with these variants would benefit from early monitoring for glaucoma in the clinic.

Just one variant analyzed in our dataset does not neatly fall into a particular category, namely, A427T. The thermal stability of A427T (47.8°C) is within the lower bound of the thermal stability of a WT-like variant but outside the range of that of a disease variant. Previously, A427T was considered mildly pathogenic because it was documented in a family with glaucoma, albeit at an age of diagnosis >40 and incomplete penetrance among family members (Faucher et al., 2002). The only experimental hints in favor of a pathogenic assignment are the lack of crystal growth in this study, previous characterization *in vitro* demonstrating a mild aggregation propensity (Hill et al., 2014) and deleterious annotations by PolyPhen and SIFT. The effect of the A427T OLF substitution should be subjected to further scrutiny.

Early monitoring and diagnosis based on genotype, combined with early intervention, could be transformative for precision clinical care of glaucoma (Lee and Mackey, 2022). Glaucoma is a heterogeneous disease, and genetic risk for glaucoma is complex and extends well beyond myocilin, but patients with the highest combination of known genetic risk variants are diagnosed on average 5 years earlier than those with fewer genetic risk variants (Fan et al., 2019). This observation indicates that genetics play a role in the disease and that prodromal screening would be valuable. The likelihood of pathogenicity for myocilin variants based on thermal stability can now be integrated with assessments of genetic risk for glaucoma even in the absence of clinical data. The ability to forecast disease phenotype based on genotype paves the way for reducing the incidence of permanent vision loss in this painless, irreversible age-onset disease.

## MATERIALS AND METHODS

### Site-directed mutagenesis

The plasmid for full-length myocilin was custom cloned by GenScript in the pcDNA 3.1 vector and includes a C-terminal FLAG tag, as reported previously (Martin et al., 2021). Mutations corresponding to T293K, R296H, L303I, V329M, S331F, E352K, K398R, A445V and A427T were produced by site-directed mutagenesis (QuikChange Lightning Mutagenesis Kit, Agilent; Table S4). The plasmids for E300K, S313F, T353I, N420Y, V449I and K500R were purchased from GENEWIZ. The plasmids for OLF variants T256M, R296H, E300K, L303I, S313F, S331L, E352K, N420Y, V449I and K500R were generated via site-directed mutagenesis (QuikChange Lightning Kit, Agilent; Table S5) in a plasmid encoding an N-terminal maltose-binding protein fusion with a tobacco etch virus (TEV) protease cleavage site (Hill et al., 2014). All sequences were verified by DNA sequencing (Eton Biosciences or Genscript).

### Cellular secretion assay

HEK293T cells (American Type Culture Collection) were grown and maintained in Dulbecco's modified Eagle medium (DMEM, Corning) supplemented with 10% fetal bovine serum (Hyclone), and 1% penicillin-streptomycin-glutamine (Gibco) at 37°C with 5% CO_2_. Cells were plated at 70-80% confluency in six-well plates 24 h prior to transfection. Plasmid transfections were carried out with Lipofectamine 2000 (Invitrogen) at a ratio of 1 µg plasmid to 2.5 µl Lipofectamine 2000 mixed in serum-free Opti-MEM (Invitrogen). Cells were transfected for 48 h prior to collection. The medium was changed to serum-free DMEM with 1% penicillin-streptomycin-glutamine 24 h post transfection.

The medium collected 48 h after transfection was treated with cOmplete protease inhibitor (Roche) and phosphatase inhibitor cocktails 2 and 3 (Sigma-Aldrich), centrifuged at 1000 ***g*** for 5 mins to remove cell debris, and then concentrated 10× with an Amicon filtration device (30 kDa molecular mass cutoff) for immunoblotting. Adherent cells were scraped and lysed in Triton X-100 lysis buffer [100 mM Tris-HCl, pH 7.4, 3 mM EGTA, 5 mM MgCl_2_, 1 mM phenylmethylsulfonyl fluoride (PMSF) and 0.5% Triton X-100] containing cOmplete protease inhibitor (Roche) and phosphatase inhibitor cocktails 2 and 3 (Sigma-Aldrich), by overnight freeze-thaw at −20°C. Cell lysates were then fractionated into soluble (supernatant) and insoluble (pellet) fractions by centrifugation at 17,000 ***g*** for 10 mins. Total protein concentrations of the medium and soluble fraction were measured by BCA assay (Pierce). Samples of media and soluble fractions for immunoblotting were then prepared with a final concentration of 1× Laemmli sample buffer containing 10% β-mercaptoethanol (v/v). Insoluble fractions were washed three times with ice-cold PBS (Gibco), resuspended in 300 μl of 2× Laemmli buffer with β-mercaptoethanol and then sonicated with a rod sonicator (Qsonica Q125) for 5 mins with 10 s on/off pulses at 50% amplitude). Insoluble samples were boiled for 30 mins at 95°C, and the media and soluble samples were boiled for 5 mins at 95°C prior to immunoblotting. Two independent experiments were conducted in analytical duplicates.

### Immunoblotting

Samples were loaded into 12% stain-free Tris-glycine SDS-polyacrylamide gels prepared in house. Gels were transferred onto polyvinylidene difluoride (PVDF) membranes (Bio-Rad) and blocked at room temperature for 1 h in 5% milk. The following primary antibodies (Table S6) were diluted 1:1000 in 1% milk and incubated overnight: anti-myocilin (MAB3446, R&D Systems), anti-β-actin (4970, Cell Signaling Technology) and anti-FLAG (F3165, Sigma Millipore). After primary antibody incubation, PVDF membranes were washed three times with PBS containing 0.1% Tween-20. The following secondary antibodies were diluted 1:2500 in 1% milk and incubated on membranes for 1 h: Starbright Blue 520 goat anti-mouse IgG and Starbright blue 700 goat anti-rabbit IgG (Bio-Rad). Images were captured with a ChemiDoc MP imaging System (Bio-Rad). Quantification of western blots was done with ImageLab software (Bio-Rad).

### Immunocytochemistry

Glass microscope coverslips (12 mm diameter, Thermo Fisher Scientific) were soaked for 30 min in 1% gelatin (Sigma-Aldrich) diluted in PBS. The solution was aspirated and allowed to air dry for 1 h in 24-well tissue-culture plates. HEK293T cells were plated at 70-80% confluency onto treated coverslips and grown overnight as described above. Cells were transfected with plasmids in serum-containing media as described above. Cells were washed 48 h post transfection with PBS and fixed in 10% formalin (Fisher Healthcare) for 30 min at room temperature and washed twice with PBS. Cells were then permeabilized with 1× assay buffer containing 0.5% Triton X-100 and 3 mM EDTA, supplied in the Proteostat Aggresome detection kit (Enzo Life Sciences, 51035-K100) and blocked overnight with 2% bovine serum albumin (BSA) in PBS. Primary antibodies [rabbit calnexin polyclonal antibody (Invitrogen, PA5-34754) and mouse monoclonal anti-FLAG (DYKDDDDK; Cell Signaling Technology, 8146)] for myocilin detection, each diluted 1:200, were prepared in 0.1% BSA in PBS and incubated for 3 h at room temperature. After primary antibody incubation, the coverslips were washed three times with PBS. Secondary antibodies [anti-rabbit Alexa Fluor 488 (Thermo Fisher Scientific, A-11034) and anti-mouse Cy5 (Thermo Fisher Scientific, A10524)] were diluted 1:1000 in 0.1% BSA in PBS and incubated for 45 min at room temperature. After secondary antibody incubation, the coverslips were washed again three times with PBS. Lastly, Proteostat and Hoechst 33342 (Enzo Life Sciences, 51035-K100) were diluted 1:2000 and 1:1000, respectively, in 1× assay buffer and incubated for 30 min at room temperature. The coverslips were washed and mounted using Antifade Gold Mounting Reagent (Invitrogen) and allowed to cure for 24 h. Fluorescence images were captured using a laser scanning confocal microscope (Zeiss LSM 700 with AxioObserver and 63×1.4 oil immersion objective).

### Colocalization analysis

Colocalization measurements were calculated using Pearson's coefficient within ImageJ (National Institutes of Health) using the JACoP plugin (Bolte and Cordelières, 2006). Statistical analysis of colocalization was performed using three independent fields of view per coverslip with one-way ANOVA and Dunnett's post hoc test using GraphPad Prism v9.

### OLF expression and purification

OLF variants were recombinantly expressed and purified as described previously (Donegan et al., 2015) with minor modifications. Briefly, Rosetta-gami 2(DE3) cells (Novagen) transformed with plasmids were grown in Superior broth (US Biological) supplemented with 60 µg/ml of ampicillin and 34 µg/ml of chloramphenicol. Cultures were induced at an OD600 of 1.5 at 18°C with 0.5 mM isopropyl-β-D-thiogalactopyranoside and 100 mM CaCl_2_, and cells were allowed to grow for 16 h. The cells were pelleted, flash frozen in liquid nitrogen and stored at −80°C. WT, T256M, R296H, L303I, S313F, S331L, E352K, V449I and K500R variants were purified by amylose affinity chromatography and size-exclusion chromatography as reported previously for WT OLF (Donegan et al., 2012). E300K and N420Y were cleaved by TEV protease at 4°C. Purity was assessed by standard 12% SDS-PAGE analysis with either Coomassie staining or using stain-free gels visualized with the Bio-Rad ChemiDoc MP imaging System.

### Thermal stability measurements

The thermal stability of OLF variants was measured by differential scanning fluorimetry (Burns et al., 2010). Briefly, purified OLF or MBP-OLF variants (1 µM) in 10 mM HEPES, pH 7.5, 200 mM NaCl were mixed with 5× SYPRO Orange dye (Invitrogen). For MBP-OLF variants (MBP-OLF^E300K^ and MBP-OLF^S313F^), 50 mM maltose was added to stabilize MBP outside the range of OLF (Burns et al., 2010). For assessing calcium binding, CaCl_2_ was added to the reaction mixture to a final concentration of 10 mM. The reactions were loaded into 96-well optical plates and sealed with optical film (Applied Biosystems). SYPRO Orange fluorescence was monitored as a function of temperature in an Applied Biosystems Step One Plus RT-PCR system with fixed excitation at 480 nm and a carboxy-X-rhodamine 610 nm emission filter. Thermal melts were conducted in triplicate from 25 to 95°C with a 1°C/min gradient. Fluorescence data were blank subtracted and fitted with a Boltzmann sigmoid in GraphPad Prism v9 to determine the midway point of unfolding, the melting temperature (T_m_).

### Circular dichroism

Far-UV CD and near-UV CD were performed as reported previously (Hill et al., 2014) to assess secondary and tertiary structure of variant OLFs. All measurements were acquired on a Jasco J-810 or J-1100 polarimeter. For far-UV CD, 7-10 µM OLF samples in gel filtration buffer (10 mM Na_2_HPO_4_, 10 mM KH_2_PO_4_, 200 mM NaCl, pH 7.2) were used. Spectra were acquired at 4°C with ten averaged scans from 300 to 200 nm at a 500 nm/min scan rate, using a 0.1 cm cuvette. Data were blank subtracted ad converted to mean residue ellipticity, *θ*=(*M*_res_×*θ*_obs_)/(10×*d*×*c*), where *M*_res_= 112.9 is the mean residue mass calculated for OLF, *θ*_obs_ is the observed ellipticity (degrees) at a given wavelength, *d* is the path length (cm) and *c* is the protein concentration (g/ml). Near-UV CD experiments were conducted with OLF variants in gel filtration buffer, pH 7.2, at a final concentration of 30-50 µM. For urea experiments, OLF was unfolded in the corresponding urea concentration for at least 1 h at 4°C prior to CD spectrum acquisition. Scans were taken at 4°C and measured from 320 to 250 nm with a scan rate of 50 nm/min in a 0.1 cm cuvette. Each measurement was the average of ten scans and converted to mean residue ellipticity.

### Crystallization and structure determination

Purified OLF variants (in 10 mM HEPES, pH 7.2 with 200 mM NaCl) were concentrated to 5-10 mg/ml. Crystals were grown by the hanging-drop method by equilibration against a reservoir containing 8-10% PEG 8000 and 0.05-0.1 M MgCl_2_. Crystals were cryo-cooled in a solution containing their respective reservoir solution supplemented with 30% glycerol. X-ray diffraction datasets were collected at the Advanced Photon Source, Argonne National Labs beamlines, Southeast Regional Collaborative Access Team (SER-CAT) 22-ID (for T293K, L303I, V329M, S331L, E352K, T353I, K398R and A445V) or 22-BM (for K500R), or at the Advanced Light Source, Lawrence Berkeley National Labs beamlines, Berkeley Center for Structural Biology (BCSB) BL-5.0.2 (for R296H). Data were processed using HKL-2000 (Otwinowski and Minor, 1997). Structures were solved by molecular replacement using Phaser (McCoy, 2007), using WT OLF (PDB ID: 4WXQ) as the search model. The models were iteratively refined using Coot (Emsley et al., 2010) and Phenix.refine (Afonine et al., 2012).

### Statistical analysis of thermal stability and age of glaucoma diagnosis

Age at diagnosis was curated from the literature (Table S3). Individual ages reported for a given variant were averaged together and plotted as the mean±s.d. versus mean T_m_±s.d., the raw values of which were obtained from measurements reported by us in the literature (Donegan et al., 2012, 2015) or measured in this study. Ages reported as a range were included as only the two extreme values. Data for variants in publications in which only average ages were reported were not combined with data from other studies in which individual ages were available. For WT, the average age at diagnosis and s.d. for glaucoma reported in Fan et al. (2019) was used. The average of each cluster was plotted as the average age±s.d. versus average T_m_±s.d. WT-like variants and disease variants segregated into two clusters. Significance was evaluated by a Student's unpaired two-tailed *t*-test. Linear regression and statistical analyses were calculated in GraphPad Prism v9.

## Supplementary Material

10.1242/dmm.049816_sup1 Supplementary information
