# Supplementary material for: Quantitative differentiation of benign and misfolded glaucoma-causing myocilin variants on the basis of protein thermal stability
Source: Dis Model Mech. 2023 Jan 13;16(1):dmm049816. doi: 10.1242/dmm.049816 (PMC9844228; doi:10.1242/dmm.049816)
Supplement: Supplementary information [file dmm-16-049816-s1.pdf]

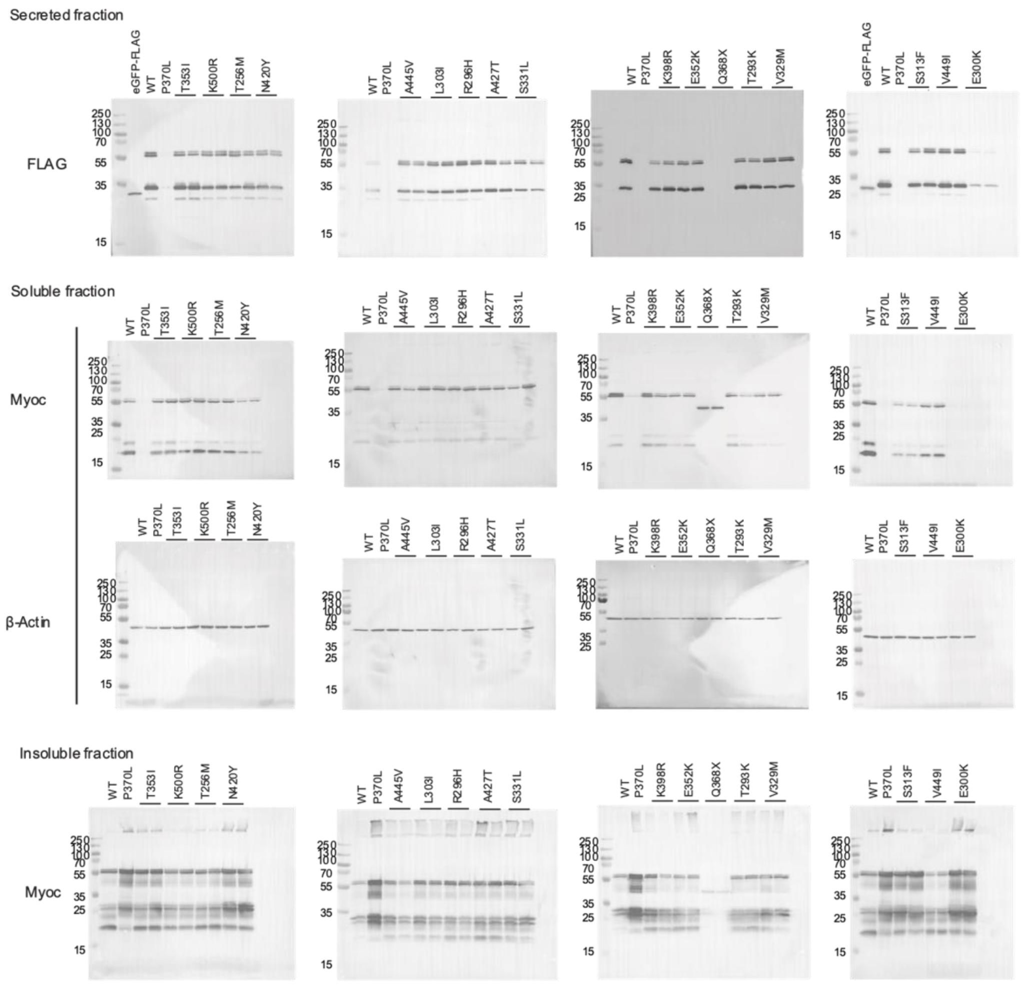

Fig. S1. Full Western blots accompanying Figure 2A. Technical replicates presented for a single experiment. Blot is representative of two independent experiments. eGFP-FLAG is a plasmid encoding for enhanced green fluorescent protein with a C-terminal tag (no myocilin).

Disease Models & Mechanisms • Supplementary information

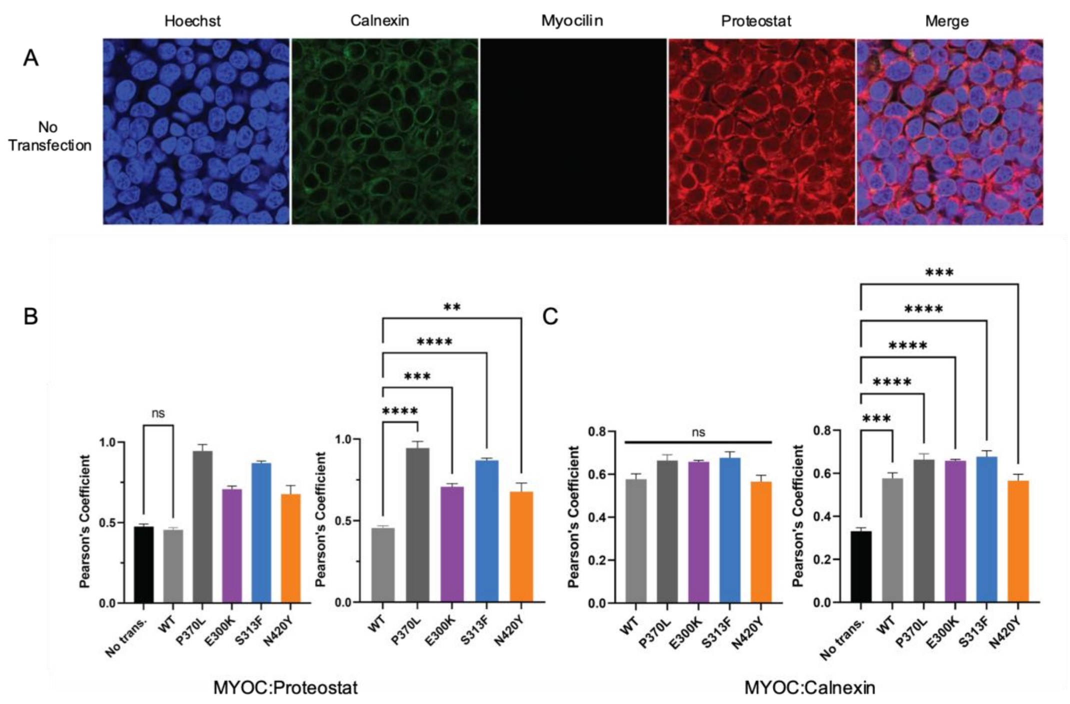

Fig. S2. Confocal images for vehicle control accompanying Figure 2C and additional statistics accompanying Figure 2D. (A) Confocal imaging of vehicle treated HEK293T cells. (B) Colocalization of myocilin and Proteostat showing no significant difference between colocalization of no transfection control and WT (left panel), whereas Proteostat staining for P370L, E300K, S313F, and N420Y are all significantly higher than WT (right panel). (C) Colocalization of myocilin and the ER marker calnexin shows similar levels of colocalization across all MYOC samples (left panel), which are statistically significant over no transfection control (right panel). For B, C statistics were calculated for n=3 technical replicate fields of view. One-way analysis of variance (ANOVA) was used for analysis using Tukey's multiple comparisons test for post-analysis. NS, not significant; * p<0.01, ** p< 0.001, *** p< 0.0001, **** p< 0.00001.

Disease Models & Mechanisms • Supplementary information

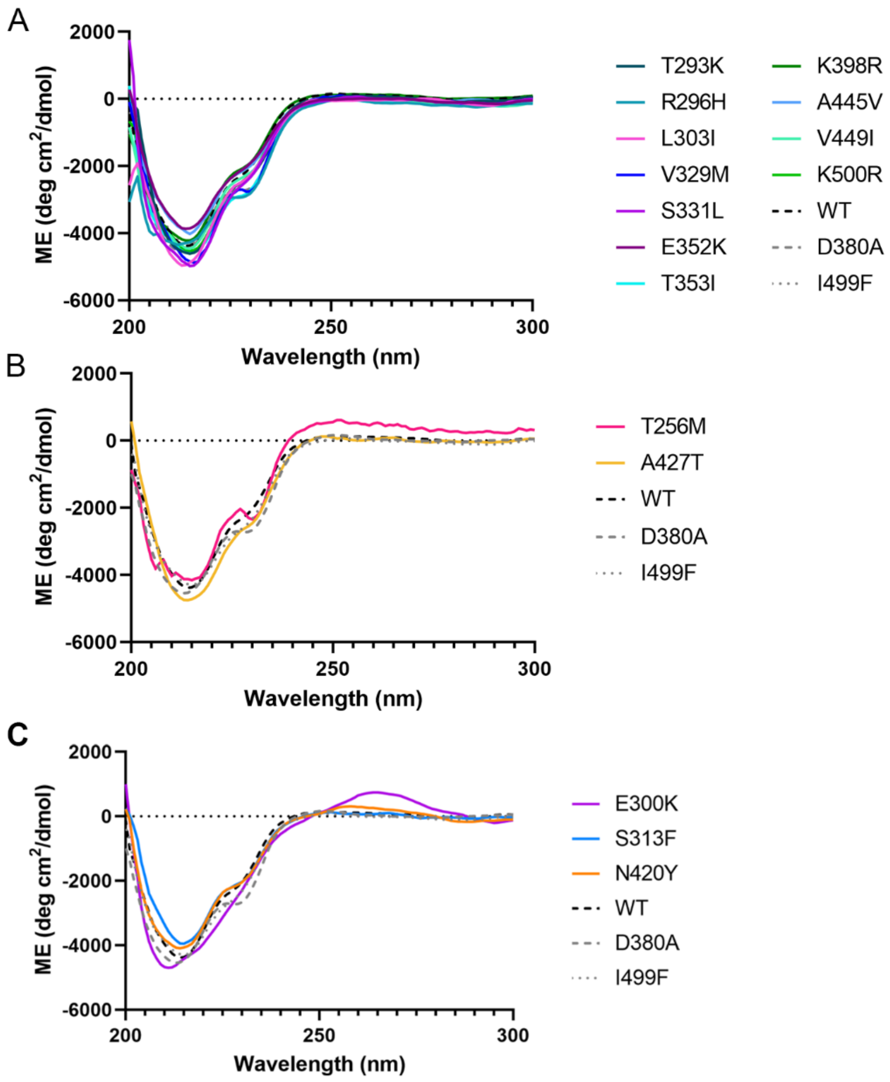

Fig. S3. Far-UV circular dichroism spectra comparing secondary structure signatures complementing data from Figure 3 and Figure 4. (A) Overlay of wild-type like variants that were structurally characterized. (B) Overlay of wild-type like variants for which structures were not obtained. (C) Overlay of candidate disease-like variants.

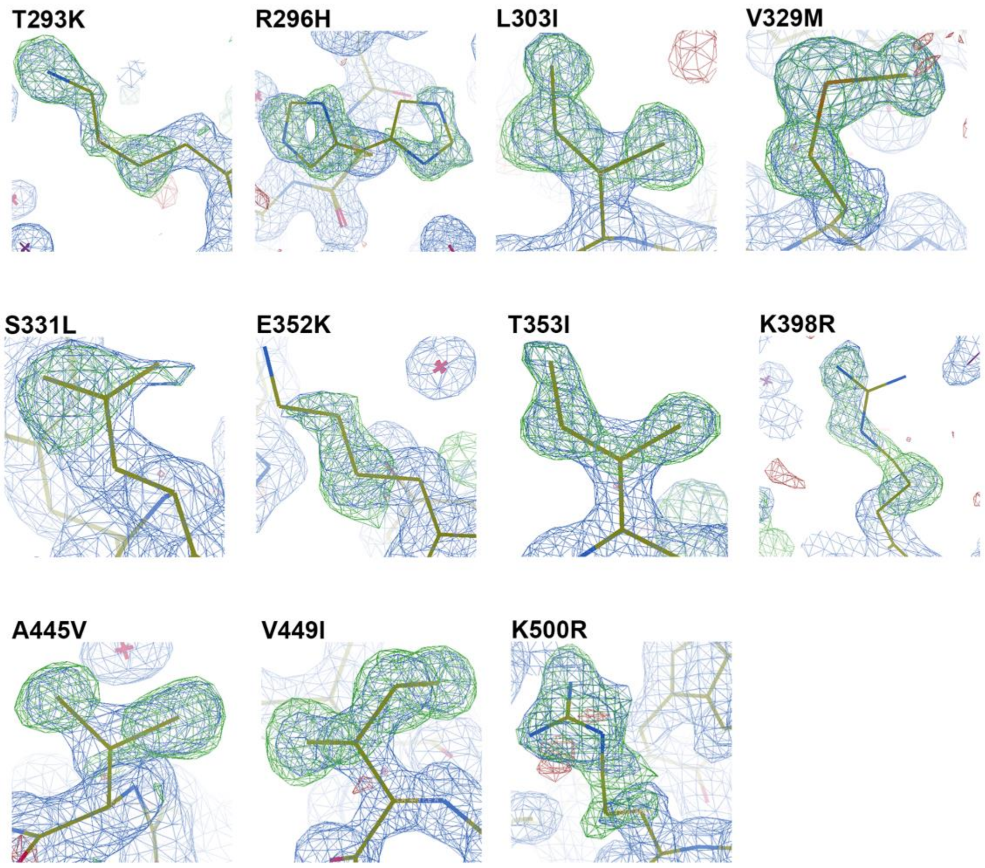

Fig. S4. Representative electron density maps for each of mutant structure. Blue mesh, final 2Fo-Fc electron density map contoured at 1σ. Green mesh, Fo-Fc difference map calculated from phases after the existing residue was replaced with alanine.

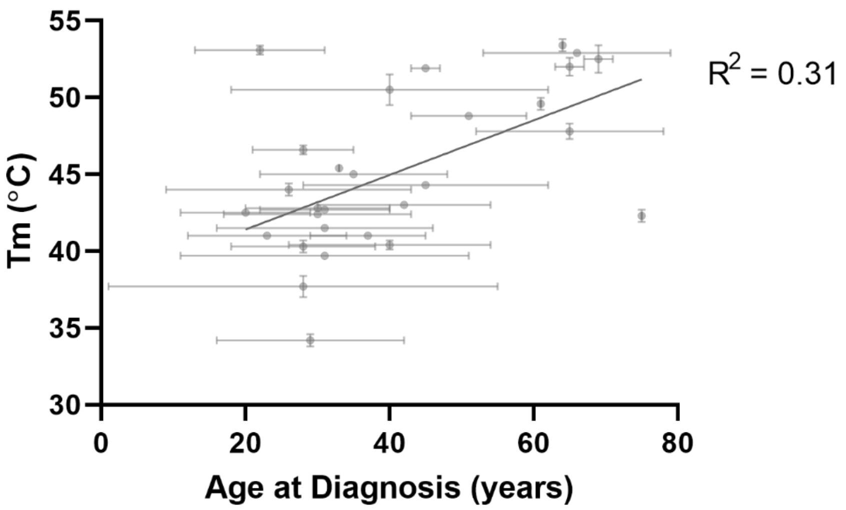

Fig. S5. Thermal stability and age at diagnosis are not well correlated by linear regression.

Table S1. OLF mutations in gnomAD with allele counts < 10, presented in increasing allele count.

HGVS Consequence	Transcript Consequence	Allele Count	Allele Frequency
p.Ile360Leu	c.1078A>C	1	3.2E-05
p.Pro370Ser	c.1108C>T	1	3.2E-05
p.Tyr335Phe	c.1004A>T	1	3.2E-05
p.Tyr301His	c.901T>C	1	3.2E-05
p.Leu403Pro	c.1208T>C	1	3.2E-05
p.Ile421Val	c.1261A>G	1	3.2E-05
p.Met504Thr	c.1511T>C	1	3.2E-05
p.Arg470Leu	c.1409G>T	1	3.2E-05
p.Thr457Ile	c.1370C>T	1	3.2E-05
p.Val251Ile	c.751G>A	1	4.1E-06
p.Thr256Ala	c.766A>G	1	4.1E-06
p.Ala260Val	c.779C>T	1	4.1E-06
p.Thr262Lys	c.785C>A	1	4.1E-06
p.Arg296Leu	c.887G>T	1	4.0E-06
p.Ser305Arg	c.913A>C	1	4.0E-06
p.Val295Ile	c.883G>A	1	4.0E-06
p.Ser305Arg	c.915C>A	1	4.0E-06
p.Val295Asp	c.884T>A	1	4.0E-06
p.Gln297Arg	c.890A>G	1	4.0E-06
p.Met308Thr	c.923T>C	1	4.0E-06
p.Gly268Asp	c.803G>A	1	4.0E-06
p.Pro321Ser	c.961C>T	1	4.0E-06
p.Thr293Ala	c.877A>G	1	4.0E-06
p.Pro312Ser	c.934C>T	1	4.0E-06
p.Asp289Glu	c.867C>A	1	4.0E-06
p.Arg287Lys	c.860G>A	1	4.0E-06
p.Met271Lys	c.812T>A	1	4.0E-06
p.Thr285Pro	c.853A>C	1	4.0E-06
p.Thr284Ala	c.850A>G	1	4.0E-06
p.Pro274Leu	c.821C>T	1	4.0E-06
p.Ile317Lys	c.950T>A	1	4.0E-06
p.Glu283Gly	c.848A>G	1	4.0E-06
p.Lys275Thr	c.824A>C	1	4.0E-06

Disease Models & Mechanisms • Supplementary information

p.Gly326Ser	c.976G>A	1	4.0E-06
p.Lys500Thr	c.1499A>C	1	4.0E-06
p.Tyr330Ser	c.989A>C	1	4.0E-06
p.Ala339Ser	c.1015G>T	1	4.0E-06
p.Thr496Ser	c.1486A>T	1	4.0E-06
p.Gln337His	c.1011G>T	1	4.0E-06
p.Leu334Val	c.1000C>G	1	4.0E-06
p.Thr351Ala	c.1051A>G	1	4.0E-06
p.Met494Lys	c.1481T>A	1	4.0E-06
p.Asn491Ser	c.1472A>G	1	4.0E-06
p.Ile345Thr	c.1034T>C	1	4.0E-06
p.Leu381Ser	c.1142T>C	1	4.0E-06
p.Lys358Glu	c.1072A>G	1	4.0E-06
p.Lys484Gln	c.1450A>C	1	4.0E-06
p.His366Arg	c.1097A>G	1	4.0E-06
p.Ala397Asp	c.1190C>A	1	4.0E-06
p.Pro481Ser	c.1441C>T	1	4.0E-06
p.Gln184Lys	c.550C>A	1	4.0E-06
p.Ile401Thr	c.1202T>C	1	4.0E-06
p.Ile401Leu	c.1201A>C	1	4.0E-06
p.Met476Leu	c.1426A>T	1	4.0E-06
p.Leu413Val	c.1237C>G	1	4.0E-06
p.Pro466Ser	c.1396C>T	1	4.0E-06
p.Glu418Lys	c.1252G>A	1	4.0E-06
p.Gly458Cys	c.1372G>T	1	4.0E-06
p.Gly456Arg	c.1366G>C	1	4.0E-06
p.Glu418Gly	c.1253A>G	1	4.0E-06
p.Gly456Asp	c.1367G>A	1	4.0E-06
p.Gly434Ser	c.1300G>A	1	4.0E-06
p.Thr419Ala	c.1255A>G	1	4.0E-06
p.Ala452Pro	c.1354G>C	1	4.0E-06
p.Asp446His	c.1336G>C	1	4.0E-06
p.Phe430Ser	c.1289T>C	1	4.0E-06
p.Gly252Glu	c.755G>A	2	8.3E-06
p.Tyr267Cys	c.800A>G	2	8.0E-06
p.Asp289Asn	c.865G>A	2	8.0E-06
p.Arg272Gln	c.815G>A	2	8.0E-06
p.Trp286Arg	c.856T>C	2	8.0E-06
p.Thr281Ala	c.841A>G	2	8.0E-06

p.Ala327Thr	c.979G>A	2	8.0E-06
p.Lys503Glu	c.1507A>G	2	8.0E-06
p.Thr377Ala	c.1129A>G	2	8.0E-06
p.Glu357Lys	c.1069G>A	2	8.0E-06
p.Gly364Ser	c.1090G>A	2	8.0E-06
p.Gly244Arg	c.730G>A	2	8.0E-06
p.Ala397Val	c.1190C>T	2	8.0E-06
p.Ser475Gly	c.1423A>G	2	8.0E-06
p.Leu413Phe	c.1237C>T	2	8.0E-06
p.Thr416Asn	c.1247C>A	2	8.0E-06
p.Ala447Val	c.1340C>T	2	8.0E-06
p.Gly456Val	c.1367G>T	2	8.0E-06
p.Asn450Ser	c.1349A>G	2	8.0E-06
p.Thr443Ile	c.1328C>T	2	8.0E-06
p.Ser313Cys	c.938C>G	2	7.1E-06
p.Leu255Val	c.763C>G	3	1.2E-05
p.Thr256Arg	c.767C>G	3	1.2E-05
p.Thr293Met	c.878C>T	3	1.2E-05
p.Pro321Thr	c.961C>A	3	1.2E-05
p.Leu334Pro	c.1001T>C	3	1.2E-05
p.Arg342Gly	c.1024A>G	3	1.2E-05
p.Gly387Asp	c.1160G>A	3	1.2E-05
p.Asp395Asn	c.1183G>A	3	1.2E-05
p.Arg470His	c.1409G>A	3	1.2E-05
p.Met476Arg	c.1427T>G	3	1.2E-05
p.Gln424His	c.1272G>C	3	1.2E-05
p.Gly252Ala	c.755G>C	3	1.1E-05
p.Arg296Cys	c.886C>T	4	1.6E-05
p.Ala339Thr	c.1015G>A	4	1.6E-05
p.Gly399Val	c.1196G>T	4	1.6E-05
p.Asn469Ser	c.1406A>G	4	1.6E-05
p.Leu303Phe	c.907C>T	4	1.4E-05
p.Ser333Asn	c.998G>A	5	2.0E-05
p.Thr377Met	c.1130C>T	5	2.0E-05
p.Ala488Val	c.1463C>T	5	2.0E-05
p.Asp395Glu	c.1185T>G	5	2.0E-05
p.Gly244Val	c.731G>T	6	2.5E-05
p.Gly239Arg	c.715G>A	6	2.4E-05
p.Trp417Leu	c.1250G>T	6	2.4E-05

Disease Models & Mechanisms • Supplementary information

p.Thr438Pro	c.1312A>C	6	2.4E-05
p.Arg470Cys	c.1408C>T	6	2.1E-05
p.Thr285Met	c.854C>T	7	2.8E-05
p.Thr325Met	c.974C>T	7	2.8E-05
p.Thr351Asn	c.1052C>A	7	2.8E-05
p.Tyr471Cys	c.1412A>G	7	2.8E-05
p.Asp294Ala	c.881A>C	7	2.5E-05
p.Asp446Tyr	c.1336G>T	8	2.8E-05
p.Val439Ile	c.1315G>A	9	3.6E-05
p.Arg422Cys	c.1264C>T	9	3.2E-05
p.Arg422His	c.1265G>A	9	3.2E-05

Disease Models & Mechanisms • Supplementary information

Table S2. Crystallographic and refinement statistics.

	T293K (PDB code 7SJU)	R296H (PDB code 7SKE)	L303I (PDB code 7SJW)	V329M (PDB code 7SKG)	S331L (PDB code 7SKD)	E352K (PDB code 7SIJ)	T353I (PDB code 7SJV)	K398R (PDB code 7SJT)	A445V (PDB code 7SKF)	V449I (PDB code 7T8D)	K500R (PDB code 7SIB)
Resolution range	35.6 - 1.39 (1.44 - 1.39)	35.58 - 1.24 (1.28 - 1.24)	35.31 - 1.38 (1.43 - 1.38)	35.69 - 1.33 (1.38 - 1.33)	35.13 - 1.71 (1.77 - 1.71)	37.26 - 1.54 (1.60 - 1.54)	35.24 - 1.39 (1.44 - 1.39)	35.68 - 1.54 (1.60 - 1.54)	33.28 - 1.28 (1.33 - 1.28)	35.69 - 1.38 (1.43 - 1.38)	35.26 - 1.78 (1.84 - 1.78)
Space group	P 1 21 1	P 1 21 1	P 1 21 1	P 1 21 1	P 1 21 1	P 1 21 1	P 1 21 1	P 1 21 1	P 1 21 1	P 1 21 1	P 1 21 1
Unit cell dimensions	49.38 50.60 50.43 90 96.75 90	49.24 50.72 50.36 90 97.67 90	49.33 50.89 50.50 90 96.40 90	49.48 50.73 50.51 90 96.22 90	49.18 50.60 50.28 90 97.15 90	49.34 50.73 50.49 90 96.60 90	49.37 50.70 50.64 90 96.85 90	49.31 50.56 50.67 90 96.38 90	49.35 50.65 50.63 90 96.49 90	49.41 50.87 50.46 90 97.01 90	49.30 50.78 50.65 90 96.31 90
Total reflections	311930 (30547)	386854 (21566)	297258 (26062)	330027 (30262)	137573 (4266)	233247 (21930)	293471 (22870)	82996 (8623)	392203 (37495)	335197 (31675)	134814 (10759)
Unique reflections	48219 (4536)	67993 (5562)	50211 (4765)	56446 (5575)	22613 (1000)	36260 (3465)	49251 (4798)	34661 (3518)	62664 (6142)	50378 (4924)	23925 (2286)
Multiplicity	6.5 (6.7)	5.7 (3.9)	5.9 (5.5)	5.8 (5.4)	6.1 (4.3)	6.4 (6.3)	6.0 (4.8)	2.4 (2.5)	6.3 (6.1)	6.7 (6.3)	5.6 (4.7)
Completeness (%)	97.01 (92.08)	97.53 (80.39)	97.81 (93.83)	98.70 (98.03)	80.84 (37.78)	98.57 (95.58)	98.31 (96.03)	94.18 (96.91)	98.42 (96.74)	98.68 (97.45)	99.60 (96.70)
Mean I/sigma(I)	26.38 (10.21)	12.59 (1.78)	25.30 (7.03)	23.02 (5.58)	10.17 (1.93)	22.48 (8.21)	16.62 (3.72)	19.54 (9.05)	25.72 (8.92)	17.82 (6.52)	18.17 (6.03)
Wilson B-factor	8.08	10.95	9.11	9.64	18.33	10.2	6.92	15.35	8.14	9.74	8.88
R-merge	0.068 (0.19)	0.073 (0.48)	0.12 (0.47)	0.087 (0.50)	0.11 (0.33)	0.051 (0.15)	0.12 (0.56)	0.037 (0.14)	0.047 (0.16)	0.066 (0.41)	0.075 (0.21)
R-meas	0.074 (0.20)	0.080 (0.55)	0.13 (0.52)	0.095 (0.55)	0.12 (0.37)	0.055 (0.17)	0.13 (0.62)	0.047 (0.18)	0.052 (0.17)	0.072 (0.45)	0.083 (0.23)
CC1/2	1 (0.98)	1 (0.84)	0.99 (0.91)	1 (0.89)	0.99 (0.93)	1 (0.99)	1 (0.89)	1 (0.94)	1 (0.98)	1 (0.94)	1 (0.95)
Reflections used in refinement	48217 (4536)	67979 (5558)	50098 (4762)	56435 (5568)	21500 (999)	36254 (3462)	49240 (4795)	34661 (3517)	62662 (6142)	50497 (4924)	23923 (2286)

Disease Models & Mechanisms • Supplementary information

Reflections used for R-free	1992 (183)	2005 (155)	2023 (189)	2002 (204)	1981 (93)	2004 (193)	1987 (191)	2014 (204)	1987 (189)	2003 (199)	1991 (185)
R-work	0.16 (0.17)	0.17 (0.28)	0.16 (0.19)	0.17 (0.23)	0.23 (0.39)	0.16 (0.16)	0.17 (0.20)	0.15 (0.16)	0.16 (0.18)	0.16 (0.21)	0.15 (0.16)
R-free	0.18 (0.21)	0.19 (0.31)	0.18 (0.21)	0.19 (0.28)	0.29 (0.42)	0.18 (0.20)	0.19 (0.22)	0.17 (0.19)	0.16 (0.20)	0.18 (0.21)	0.18 (0.21)
Number of non-hydrogen atoms	2349	2322	2396	2442	2196	2349	2266	2299	2451	2360	2369
macromolecules	2124	2175	2173	2178	2110	2130	2138	2120	2202	2136	2121
ligands	14	26	8	14	2	8	2	2	8	8	8
solvent	211	121	215	250	84	211	126	177	241	216	240
Protein residues	259	259	259	259	259	259	259	259	259	259	259
RMS(bonds)	0.005	0.004	0.005	0.005	0.007	0.005	0.005	0.008	0.005	0.005	0.006
RMS(angles)	0.9	0.85	0.85	0.87	0.91	0.88	0.87	1.05	0.89	0.89	0.86
Ramachandran favored (%)	95.72	96.5	96.5	96.89	95.33	96.11	96.5	97.28	96.11	96.5	96.5
Ramachandran allowed (%)	4.28	3.5	3.5	3.11	4.67	3.89	3.5	2.72	3.89	3.5	3.5
Ramachandran outliers %	0	0	0	0	0	0	0	0	0	0	0

Disease Models & Mechanisms • Supplementary information

Rotamer outliers (%)	0	1.27	0.84	0.84	1.75	0	0	0.87	1.24	0.86	0
Clashscore	1.18	2.98	2.78	2.07	3.6	0.24	0.95	0.95	4.34	1.65	3.57
Average B-factor	11.58	14.46	13.5	13.44	24.55	13.35	10.41	18.41	12.01	12.95	11.27
macromolecules	10.49	13.89	12.36	12.06	24.48	12.28	10.02	17.58	10.8	11.85	10.04
ligands	24.16	25.28	22.76	25.34	18.17	23.64	4.62	11.65	21.72	18.54	22.84
solvent	21.73	22.26	24.64	24.82	26.38	23.7	17.2	28.48	22.69	23.62	21.76

Disease Models & Mechanisms • Supplementary information

Table S3. Data used in analysis of Tm versus age at diagnosis.

Variant	Age at Diagnosis	Ref	Tm (°C)[a]	Ref
G246R	11, 28<br>*Average:* 20 ± 9	(Adam et al., 1997)	42.4, 42.7, 42.3<br>*Average:* 42.5 ± 0.2	(Donegan et al., 2012)
G252R	31, 36, 36, 42, 50, 57, 58, 60<br>26<br>29, 38<br>*Average:* 42 ± 12	(Hewitt et al., 2007)<br><br>(Rozsa et al., 1998; Shimizu et al., 2000)<br>(Vincent et al., 2002)	43.1, 42.8, 43.0<br><br><br><br>*Average:* 43.0 ± 0.1	(Donegan et al., 2012)
T256M	46<br>43<br>*Average:* 45 ± 2	(Bhattacharjee et al., 2007)<br>(Banerjee et al., 2012)	52.0, 51.7, 52.0<br><br>*Average:* 52.0 ± 0.1	(this work)
R272G	32, 29, 45<br>*Average:* 37 ± 8	(Shimizu et al., 2000)	41.3, 40.9, 40.8<br>*Average:* 41.0 ± 0.2	(Donegan et al., 2012)
T293K	71<br>54<br>67<br>*Average:* 69 ± 2	(Ennis et al., 2010)<br>(Williams-Lyn et al., 2000)<br>(Faucher et al., 2002)	52.2, 52.3, 52.3, 53.5, 53.6, 53.5<br>52.4, 51.2, 51.2<br>*Average:* 52.5 ± 0.9	(Donegan et al., 2015)<br><br>(this work)
E300K	75<br>*Average:* 75 ± 0	(Pang et al., 2002)	41.8, 42.6, 42.5<br>*Average:* 42.3 ± 0.4	(this work)
E323K	19, 9, 43<br>*Average:* 26 ± 17	(Rozsa et al., 1998; Shimizu et al., 2000)	43.8, 44.6, 43.7<br>*Average:* 44.0 ± 0.4	(Donegan et al., 2012)
V329M	30, 70<br>20<br><br>*Average:* 40 ± 22	(Shimizu et al., 2000)<br>(Svidnicki et al., 2018)	49.8, 49.8, 49.4, 49.8, 49.8, 50.6<br>52.3, 52.0, 51.1<br>*Average:* 50.5 ± 1.0	(Donegan et al., 2015)<br><br>(this work)
S331L	61<br>*Average:* 61 ± 0	(Banerjee et al., 2012)	50.1, 49.6, 49.1<br>*Average:* 49.6 ± 0.4	(this work)
E352K	51, 60<br>41<br>*Average:* 51 ± 8	(Williams-Lyn et al., 2000)<br>(Faucher et al., 2002)	48.7, 48.8, 48.8<br><br>*Average:* 48.8 ± 0.0	(this work)
T353I	29<br>12<br>14<br>32<br>*Average:* 22 ± 9	(Zhou et al., 2013)<br>(Park et al., 2016)<br>(Rose et al., 2011)<br>(Bhattacharjee et al., 2007)	52.9, 53.1, 53.1, 52.9, 52.8, 53.7<br>53.3, 53.2, 53.1<br><br>*Average:* 53.1 ± 0.3	(Donegan et al., 2015)<br><br>(this work)
G364V	22, 48<br>*Average:* 35 ± 13	(Alward et al., 1998)	45.1, 44.7, 45.3<br>*Average:* 45.0 ± 0.2	(Donegan et al., 2012)
G367R	45<br>45<br>32	(Taniguchi et al., 2000)<br>(Suzuki et al.,	42.8, 42.6, 42.8	(Donegan et al., 2012)

Disease Models & Mechanisms • Supplementary information

	51, 27, 28, 28, 30, 30, 42, 40, 38<br>14, 13, 20<br>38, 30, 46, 20, 25<br>34, 39, 25<br>32, 31, 22<br>30<br>*Average:* 31 ± 9	1997)<br>(Kanagavalli et al., 2003)<br>(Iliev et al., 2008)<br><br>(Souzeau et al., 2015)<br>(Chen et al., 2011)<br>(Faucher et al., 2002)<br>(Yao et al., 2018)<br>(Vincent et al., 2002)	*Average:* 42.7 ± 0.1	
T377M	10, 40<br>42<br>52<br>20, 60<br>47<br>66, 25, 29, 25, 14, 22<br>68, 72, 74, 52, 60, 82, 56, 55, 71, 74, 71, 16, 36<br>75, 67, 60, 57, 54, 36, 34, 36, 39, 46, 43, 32, 70, 58, 67<br>34, 26, 25<br>70, 56, 38, 41, 36, 42, 54, 30, 40, 31, 30, 31, 44, 36, 37, 35, 32, 32, 57, 52, 32, 47<br>34, 44<br>*Average:* 45 ± 17	(Zgaga et al., 2008)<br>(Wiggs et al., 1998)<br>(Kanagavalli et al., 2003)<br>(Alward et al., 1998)<br>(Liu et al., 2012)<br>(Puska et al., 2005)<br>(Wirtz et al., 2008)<br><br>(Kitsos et al., 2010; Petersen et al., 2006)<br><br>(Young et al., 2012)<br>(Mackey et al., 2003)<br><br>(Shimizu et al., 2000)	44.5, 44.1, 44.3<br>*Average:* 44.3 ± 0.2	(Donegan et al., 2012)
D380A	21, 35<br>*Average:* 28 ± 7	(Stoilova et al., 1998)	46.4, 47.0, 46.3<br>*Average:* 46.6 ± 0.3	(Donegan et al., 2012)
K398R	64<br>*Average:* 64 ± 0	(Vázquez et al., 2009)	53.8, 52.9, 53.5<br>*Average:* 53.4 ± 0.4	(Donegan et al., 2012)
K423E	9<br>50, 30, 27<br>44, 16, 36, 11<br>38, 29<br>*Average*: 29 ± 13	(Svidnicki et al., 2018)<br>(Bruttini et al., 2003)<br>(Morissette et	33.9, 33.9, 34.7<br>*Average*: 34.2 ± 0.4	(Donegan et al., 2012)

Disease Models & Mechanisms • Supplementary information

		al., 1998)<br>(Faucher et al., 2002)		
V426F	26, 16, 46<br>*Average:* 31 ± 15	(Rozsa et al., 1998)	41.5, 41.5, 41.4<br>*Average:* 41.5 ± 0.0	(Donegan et al., 2012)
A427T	73<br>46, 76<br>*Average:* 65 ± 13	(Faucher et al., 2002)<br>(Bhattacharjee et al., 2007)	47.8, 47.7<br>47.8, 48.7, 47.0<br>*Average:* 47.8 ± 0.5	(Donegan et al., 2012)<br>(this work)
C433R	15, 35<br>30<br>17, 58, 40, 49, 47, 32, 43, 55, 53<br>*Average:* 40 ± 14	(Vasconcellos et al., 2000)<br>(Svidnicki et al., 2018)<br>(Povoa et al., 2006)	40.5, 40.6, 40.0<br>*Average:* 40.4 ± 0.3	(Donegan et al., 2012)
Y437H	31, 28, 32, 46, 25, 26, 28<br>16<br>8, 41<br>*Average:* 28 ± 10	(Lei et al., 2019)<br>(Wiggs et al., 1998)<br>(Alward et al., 1998)	39.8, 40.6, 40.2, 40.8, 40.5, 40.3, 39.6<br>*Average:* 40.3 ± 0.4	(Donegan et al., 2012)
A445V	63<br>67<br>*Average:* 65 ± 2	(Faucher et al., 2002)<br>(Lopez-Martinez et al., 2007)	51.5, 52.9, 51.7<br>*Average:* 52.0 ± 0.6	(this work)
I477N	4, 26<br>18, 4, 80<br>12, 41<br>*Average:* 28 ± 27	(Rozsa et al., 1998)<br>(Shimizu et al., 2000)<br>(Alward et al., 1998)	37, 37.3, 37.5, 39.1, 37.8<br>*Average:* 37.7 ± 0.7	(Donegan et al., 2012)
I477S	11, 51<br>*Average:* 31 ± 20	(Adam et al., 1997)	39.5, 39.7, 39.9<br>*Average:* 39.7 ± 0.2	(Donegan et al., 2012)
N480K	38, 25, 40, 40, 42, 40, 40, 36, 31, 36, 34, 29, 32, 37, 34, 17, 23, 32, 24, 25, 30, 25, 16, 22, 17, 16, 16, 31, 18, 13<br>40, 40, 34<br>21<br>10, 65, 21, 51, 14, 75<br>14<br>*Average:* 30 ± 13	(Mimivati et al., 2014)<br>(Hulsman et al., 2002)<br>(Brézin et al., 1998)<br>(Adam et al., 1997)<br>(Rose et al., 2011)	42.2, 42.6, 42.4<br>*Average:* 42.4 ± 0.2	(Donegan et al., 2012)
P481L	33<br>*Average:* 33 ± 0	(Faucher et al., 2002)	45.7, 45.1, 45.5<br>*Average:* 45.5 ± 0.2	(Donegan et al., 2012)

Disease Models & Mechanisms • Supplementary information

I499F	20, 40<br>*Average:* 30 ± 10	(Adam et al., 1997)	42.7, 42.9, 42.8<br>*Average:* 42.8 ± 0.1	(Donegan et al., 2012)
S502P	12, 33<br>*Average:* 23 ± 11	(Stoilova et al., 1998)	41.3, 40.8, 40.9<br>*Average:* 41.0 ± 0.2	(Donegan et al., 2012)
WT	66<br>*Average:* 66 ± 13	(Fan et al., 2019)	52.9, 53.0, 52.9<br>*Average:* 53.0 ± 0.0	(Donegan et al., 2012)

Table S4. Primers used for site-directed mutagenesis for full-length myocilin plasmid.

Variant	Forward	Reverse
T256M	5′- ctgctgttctcagc**a**tgagaggctctcctac -3′	5′- gtaggagagcctctca**t**gctgagaacagcag -3′
T293K	5′- ctggcgacatcccaactgtgtcgat-3′	5′- atcgacacagttgggatgtccgccag-3′
R296H	5′- catactcaaaaacctgg**t**ggacatccgtgccaac -3′	5′- gttggcacggatgtcc**a**ccaggtttttgagtatg -3′
L303I	5′- caggtttttgagtatgac**c**tcatcagccagtttatgcagg -3′	5′- caggtttttgagtatgac**a**tcatcagccagtttatgcagg -3′
V329M	5′- ctccccgagtacacacagcaccctg -3′	5′- cacgggtgctgtgtgtactcgggag -3′
S331L	5′- gaaatagaggctcccc**a**agtacaccacagcacc -3′	5′- ggtgctgtggtgtact**t**ggggagcctctatttc -3′
E352K	5′- ctcagccttcactgtct**t**ggtattcagctcatatc-3′	5′- gatatgagctgaatacc**a**agacagtgaaggctgag -3′
K398R	5′- cgatgaggccagaccattgtcctctc -3′	5′- gagaggacaatggtctggcctcatcg -3′
A427T	5′- catccgtaagcagccttcatcatctg-3′	5′- cagatgatgaagctgcttacttacggatg-3′
A445V	5′- gcagctacacctctgctaccgtcaac -3′	5′- gttgacggtagcagaggtgtagctgc -3′

Disease Models & Mechanisms • Supplementary information

Table S5. Primers used for site-directed mutagenesis for MBP-OLF plasmid.

Variant	FWD	RVS
T256M	5′- ctgctgttctcagc**a**tgagaggctctcctac -3′	5′- gtaggagagcctctca**t**gctgagaacagcag -3′
R296H	5′- catactcaaaaacctgg**t**ggacatccgtgccaac -3′	5′- gttggcacggatgtcc**a**ccaggtttttgagtatg -3′
E300K	5′- ggctgatgaggtcatact**t**aaaaacctggcggacatc -3′	5′- gatgtccgccaggttttt**a**agtatgacctcatcagcc -3′
L303I	5′- caggtttttgagtatgac**c**tcatcagccagtttatgcagg -3′	5′- caggtttttgagtatgac**a**tcatcagccagtttatgcagg -3′
S313F	5′- ggcagtatgtgaacctta**a**aagggtagccctgc -3′	5′- gcagggctaccctt**t**taaggttcacatactgcc -3′
S331L	5′- gaaatagaggctcccc**a**agtacaccacagcacc -3′	5′- ggtgctgtggtgtact**t**ggggagcctctatttc -3′
E352K	5′- ctcagccttcactgtct**t**ggtattcagctcatatc-3′	5′- gatatgagctgaatacc**a**agacagtgaaggctgag -3′
N420Y	5′- gactgactgcttacggatgtatgtctcccaggtttg-3′	5′- caaacctgggagaca**t**acatccgtaagcagtcagtc -3′
V449I	5′- gtgtcataagcaaagttga**t**ggtagcatctgctgag -3′	5′- ctcagcagatgctacc**a**tcaactttgcttatgacac -3′
K500R	5′- gccttatcacatcttggaaagc**c**tgatgtcataagtgac -3′	5′- gtcacttatgacatca**g**gctttccaagatgtgataaggc -3′

Table S6. Antibodies used in this study.

Antibody	Species	Company	Catalog Number
		Primary	
FLAG (-DYKDDDDK)	Mouse	Sigma-Aldrich	F3165
FLAG (-DYKDDDDK)	Mouse	Cell Signaling Technology	8146
Myocilin	Mouse	R&D systems	MAB3446
B-Actin	Rabbit	Cell Signaling Technology	4970
Calnexin	Rabbit	Invitrogen	PA5-34754
		Secondary	
Starbright Blue 520	Mouse	Bio-Rad	12005866
Starbright Blue 700	Rabbit	Bio-Rad	12004161
Alexa Fluor 488	Rabbit	ThermoFisher	A-11008
Cy5	Mouse	ThermoFisher	A-10524

Disease Models & Mechanisms • Supplementary information

SI References

Adam, M. F., Belmouden, A., Binisti, P., Brézin, A. P., Valtot, F., Béchetoille, A., Dascotte, J. C., Copin, B., Gomez, L., Chaventré, A. et al. (1997). Recurrent mutations in a single exon encoding the evolutionarily conserved olfactomedin-homology domain of TIGR in familial open-angle glaucoma. *Hum Mol Genet* **6**, 2091-7.

Alward, W. L. M., Fingert, J. H., Coote, M. A., Johnson, A. T., Lerner, S. F., Junqua, D., Durcan, F. J., McCartney, P. J., Mackey, D. A., Sheffield, V. C. et al. (1998). Clinical features associated with mutations in the chromosome 1 open-angle glaucoma gene (GLC1A). *N Engl J Med* **338**, 1022-1027.

Banerjee, D., Bhattacharjee, A., Ponda, A., Sen, A. and Ray, K. (2012). Comprehensive analysis of myocilin variants in east Indian POAG patients. *Mol Vis* **18**, 1548-57.

Bhattacharjee, A., Acharya, M., Mukhopadhyay, A., Mookherjee, S., Banerjee, D., Bandopadhyay, A. K., Thakur, S. K. D., Sen, A. and Ray, K. (2007). Myocilin variants in Indian patients with open-angle glaucoma. *Arch Ophthalmol* **125**, 823-829.

Brézin, A. P., Adam, M. F., Belmouden, A., Lureau, M.-A., Chaventré, A., Copin, B., Gomez, L., de Dinechin, S. D., Berkani, M., Valtot, F. et al. (1998). Founder effect in GLC1A-linked familial open-angle glaucoma in Northern France. *Am J Med Genet* **76**, 438-445.

Bruttini, M., Longo, I., Frezzotti, P., Ciappetta, R., Randazzo, A., Orzalesi, N., Fumagalli, E., Caporossi, A., Frezzotti, R. and Renieri, A. (2003). Mutations in the myocilin gene in families with primary open-angle glaucoma and juvenile open-angle glaucoma. *Arch Ophthalmol* **121**, 1034-8.

Chen, J., Cai, S. P., Yu, W., Yan, N., Tang, L., Chen, X. and Liu, X. (2011). Sequence analysis of MYOC and CYP1B1 in a Chinese pedigree of primary open-angle glaucoma. *Mol Vis* **17**, 1431-5.

Donegan, R. K., Hill, S. E., Freeman, D. M., Nguyen, E., Orwig, S. D., Turnage, K. C. and Lieberman, R. L. (2015). Structural basis for misfolding in myocilin-associated glaucoma. *Hum Mol Genet* **24**, 2111-24.

Donegan, R. K., Hill, S. E., Turnage, K. C., Orwig, S. D. and Lieberman, R. L. (2012). The glaucoma-associated olfactomedin domain of myocilin is a novel calcium binding protein. *J Biol Chem* **287**, 43370-7.

Ennis, S., Gibson, J., Griffiths, H., Bunyan, D., Cree, A. J., Robinson, D., Self, J., MacLeod, A. and Lotery, A. (2010). Prevalence of myocilin gene mutations in a novel UK cohort of POAG patients. *Eye* **24**, 328-333.

Fan, B. J., Bailey, J. C., Igo, R. P., Jr, Kang, J. H., Boumenna, T., Brilliant, M. H., Budenz, D. L., Fingert, J. H., Gaasterland, T., Gaasterland, D. et al. (2019). Association of a primary open-angle glaucoma genetic risk score with earlier age at diagnosis. *JAMA Ophthalmol* **137**, 1190-1194.

Faucher, M., Anctil, J. L., Rodrigue, M. A., Duchesne, A., Bergeron, D., Blondeau, P., Côté, G., Dubois, S., Bergeron, J., Arseneault, R. et al. (2002). Founder TIGR/myocilin mutations for glaucoma in the Québec population. *Hum Mol Genet* **11**, 2077-90.

Disease Models & Mechanisms • Supplementary information

Hewitt, A. W., Bennett, S. L., Richards, J. E., Dimasi, D. P., Booth, A. P., Inglehearn, C., Anwar, R., Yamamoto, T., Fingert, J. H., Héon, E. et al. (2007). Myocilin Gly252Arg mutation and glaucoma of intermediate severity in Caucasian individuals. *Arch Ophthalmol* **125**, 98-104.

Hulsman, C. A., De Jong, P. T., Lettink, M., Van Duijn, C. M., Hofman, A. and Bergen, A. A. (2002). Myocilin mutations in a population-based sample of cases with open-angle glaucoma: the Rotterdam Study. *Graefes Arch Clin Exp Ophthalmol* **240**, 468-74.

Iliev, M. E., Bodmer, S., Gallati, S., Lanz, R., Sturmer, J., Katsoulis, K., Wolf, S., Trittibach, P. and Sarra, G. M. (2008). Glaucoma phenotype in a large Swiss pedigree with the myocilin Gly367Arg mutation. *Eye* **22**, 880-888.

Kanagavalli, J., Krishnadas, S. R., Pandaranayaka, E., Krishnaswamy, S. and Sundaresan, P. (2003). Evaluation and understanding of myocilin mutations in Indian primary open angle glaucoma patients. *Mol Vis* **9**, 606-14.

Kitsos, G., Petrou, Z., Grigoriadou, M., Samples, J. R., Hewitt, A. W., Kokotas, H., Giannoulia-Karantana, A., Mackey, D. A., Wirtz, M. K., Moschou, M. et al. (2010). Primary open angle glaucoma due to T377M MYOC: Population mapping of a Greek founder mutation in Northwestern Greece. *Clin Ophthalmol* **4**, 171-8.

Lei, L., Li, S., Liu, X. and Zhang, C. (2019). The clinical feature of myocilin Y437H mutation in a Chinese family with primary open-angle glaucoma. *Br J Ophthalmol* **103**, 1524-1529.

Liu, W., Liu, Y., Challa, P., Herndon, L. W., Wiggs, J. L., Girkin, C. A., Allingham, R. R. and Hauser, M. A. (2012). Low prevalence of myocilin mutations in an African American population with primary open-angle glaucoma. *Mol Vis* **18**, 2241-6.

Lopez-Martinez, F., Lopez-Garrido, M. P., Sanchez-Sanchez, F., Campos-Mollo, E., Coca-Prados, M. and Escribano, J. (2007). Role of MYOC and OPTN sequence variations in Spanish patients with primary open-angle glaucoma. *Mol Vis* **13**, 862-72.

Mackey, D. A., Healey, D. L., Fingert, J. H., Coote, M. A., Wong, T. L., Wilkinson, C. H., McCartney, P. J., Rait, J. L., de Graaf, A. P., Stone, E. M. et al. (2003). Glaucoma phenotype in pedigrees with the myocilin Thr377Met mutation. *Arch Ophthalmol* **121**, 1172-80.

Mimivati, Z., Nurliza, K., Marini, M. and Liza-Sharmini, A. (2014). Identification of MYOC gene mutation and polymorphism in a large Malay family with juvenile-onset open angle glaucoma. *Mol Vis* **20**, 714-23.

Morissette, J., Clépet, C., Moisan, S., Dubois, S., Winstall, E., Vermeeren, D., Nguyen, T. D., Polansky, J. R., Côté, G., Anctil, J. L. et al. (1998). Homozygotes carrying an autosomal dominant TIGR mutation do not manifest glaucoma. *Nat Genet* **19**, 319-21.

Pang, C. P., Leung, Y. F., Fan, B., Baum, L., Tong, W. C., Lee, W. S., Chua, J. K., Fan, D. S., Liu, Y. and Lam, D. S. (2002). TIGR/MYOC gene sequence alterations in individuals with and without primary open-angle glaucoma. *Invest Ophthalmol Vis Sci* **43**, 3231-5.

Park, J., Kim, M., Park, C. K., Chae, H., Lee, S., Kim, Y., Jang, W., Chi, H. Y., Park, H. Y. and Park, S. H. (2016). Molecular analysis of myocilin and optineurin genes in Korean primary glaucoma patients. *Mol Med Rep* **14**, 2439-48.

Petersen, M. B., Kitsos, G., Samples, J. R., Gaudette, N. D., Economou-Petersen, E., Sykes, R. e., Rust, K., Grigoriadou, M., Aperis, G., Choi, D. et al. (2006). A Large GLC1C Greek Family with a Myocilin T377M Mutation: Inheritance and Phenotypic Variability. *Invest Ophthalmol Vis Sci* **47**, 620-625.

Povoa, C. A., Malta, R. F., Rezende Mde, M., de Melo, K. F. and Giannella-Neto, D. (2006). Correlation between genotype and phenotype in primary open angle glaucoma of Brazilian families with mutations in exon 3 of the TIGR/MYOC gene. *Arq Bras Oftalmol* **69**, 289-97.

Puska, P., Lemmela, S., Kristo, P., Sankila, E. M. and Jarvela, I. (2005). Penetrance and phenotype of the Thr377Met Myocilin mutation in a large Finnish family with juvenile- and adult-onset primary open-angle glaucoma. *Ophthalmic Genet* **26**, 17-23.

Rose, R., Balakrishnan, A., Muthusamy, K., Arumugam, P., Shanmugam, S. and Gopalswamy, J. (2011). Myocilin mutations among POAG patients from two populations of Tamil Nadu, South India, a comparative analysis. *Mol Vis* **17**, 3243-53.

Rozsa, F. W., Shimizu, S., Lichter, P. R., Johnson, A. T., Othman, M. I., Scott, K., Downs, C. A., Nguyen, T. D., Polansky, J. and Richards, J. E. (1998). GLC1A mutations point to regions of potential functional importance on the TIGR/MYOC protein. *Mol Vis* **4**, 20.

Shimizu, S., Lichter, P. R., Johnson, A. T., Zhou, Z., Higashi, M., Gottfredsdottir, M., Othman, M., Moroi, S. E., Rozsa, F. W., Schertzer, R. M. et al. (2000). Age-dependent prevalence of mutations at the GLC1A locus in primary open-angle glaucoma. *Am J Ophthalmol* **130**, 165-77.

Souzeau, E., Glading, J., Ridge, B., Wechsler, D., Chehade, M., Dubowsky, A., Burdon, K. P. and Craig, J. E. (2015). Predictive genetic testing in minors for Myocilin juvenile onset open angle glaucoma. *Clin Genet* **88**, 584-8.

Stoilova, D., Child, A., Brice, G., Desai, T., Barsoum-Homsy, M., Ozdemir, N., Chevrette, L., Adam, M. F., Garchon, H. J., Pitts Crick, R. et al. (1998). Novel TIGR/MYOC mutations in families with juvenile onset primary open angle glaucoma. *J Med Genet* **35**, 989-92.

Suzuki, Y., Shirato, S., Taniguchi, F., Ohara, K., Nishimaki, K. and Ohta, S. (1997). Mutations in the TIGR gene in familial primary open-angle glaucoma in Japan. *Am J Hum Genet* **61**, 1202-4.

Svidnicki, P. V., Braghini, C. A., Costa, V. P., Schimiti, R. B., de Vasconcellos, J. P. C. and de Melo, M. B. (2018). Occurrence of MYOC and CYP1B1 variants in juvenile open angle glaucoma Brazilian patients. *Ophthalmic Genet* **39**, 717-724.

Taniguchi, F., Suzuki, Y., Shirato, S. and Araie, M. (2000). The Gly367Arg mutation in the myocilin gene causes adult-onset primary open-angle glaucoma. *Jpn J Ophthalmol* **44**, 445-8.

Vasconcellos, J. P., Melo, M. B., Costa, V. P., Tsukumo, D. M., Bassères, D. S., Bordin, S., Saad, S. T. and Costa, F. F. (2000). Novel mutation in the MYOC gene in primary open glaucoma patients. *Journal of medical genetics* **37**, 301-303.

Vázquez, M. C., Herrero, M. V. O., Bastús, M. B. and Pérez, D. V. (2009). Mutations in the third exon of the MYOC gene in Spanish patients with primary open angle glaucoma. *Ophthalmic Genet* **21**, 109-115.

Vincent, A. L., Billingsley, G., Buys, Y., Levin, A. V., Priston, M., Trope, G., Williams-Lyn, D. and Héon, E. (2002). Digenic inheritance of early-onset glaucoma: CYP1B1, a potential modifier gene. *Am J Hum Genet* **70**, 448-60.

Wiggs, J. L., Allingham, R. R., Vollrath, D., Jones, K. H., De La Paz, M., Kern, J., Patterson, K., Babb, V. L., Del Bono, E. A., Broomer, B. W. et al. (1998). Prevalence of mutations in TIGR/Myocilin in patients with adult and juvenile primary open-angle glaucoma. *Am J Hum Genet* **63**, 1549-52.

Williams-Lyn, D., Flanagan, J., Buys, Y., Trope, G. E., Fingert, J., Stone, E. M. and Héon, E. (2000). The genetic aspects of adult-onset glaucoma: a perspective from the Greater Toronto area. *Can J Ophthalmol* **35**, 12-7.

Wirtz, M. K., Konstas, A. G., Samples, J. R., Kaltsos, K., Economou, A., Dimopoulos, A., Georgiadou, I. and Petersen, M. B. (2008). Myocilin variations and familial glaucoma in Taxiarchis, a small Greek village. *Mol Vis* **14**, 774-81.

Yao, Y. H., Wang, Y. Q., Fang, W. F., Zhang, L., Yang, J. H. and Zhu, Y. H. (2018). A recurrent G367R mutation in MYOC associated with juvenile open angle glaucoma in a large Chinese family. *Int J Ophthalmol* **11**, 369-374.

Young, T. K., Souzeau, E., Liu, L., Kearns, L. S., Burdon, K. P., Craig, J. E. and Ruddle, J. B. (2012). Compound heterozygote myocilin mutations in a pedigree with high prevalence of primary open-angle glaucoma. *Mol Vis* **18**, 3064-9.

Zgaga, L., Hayward, C., Vatavuk, Z., Bencic, G., Zemunik, T., Valkovic, A., Valkovic-Antic, I., Bucan, K. and Rudan, I. (2008). High prevalence of glaucoma in Veli Brgud, Croatia, is caused by a dominantly inherited T377M mutation in the MYOC gene. *Br J Ophthalmol* **92**, 1567-8.

Zhou, X.-M., Yin, Y., Fan, N., Cheng, H.-B., Li, X.-H., Wang, Y., Yu, W.-H., Cai, S.-P. and Liu, X.-Y. (2013). Single nucleotide polymorphism of MYOC affected the severity of primary open angle glaucoma. *Int J Ophthalmol* **6**, 264-268.

Disease Models & Mechanisms • Supplementary information
